# The Association Between Emotion Regulation, Physiological Arousal, and Performance in Math Anxiety

**DOI:** 10.3389/fpsyg.2021.639448

**Published:** 2021-05-11

**Authors:** Rachel G. Pizzie, David J. M. Kraemer

**Affiliations:** ^1^Educational Neuroscience Program, Gallaudet University, Washington, DC, United States; ^2^Department of Education, Dartmouth College, Hanover, NH, United States; ^3^Department of Psychological and Brain Sciences, Dartmouth College, Hanover, NH, United States

**Keywords:** math anxiety, anxiety, reappraisal, electrodermal activity, emotion regulation

## Abstract

Emotion regulation (ER) strategies may reduce the negative relationship between math anxiety and mathematics accuracy, but different strategies may differ in their effectiveness. We recorded electrodermal activity (EDA) to examine the effect of physiological arousal on performance during different applied ER strategies. We explored how ER strategies might affect the decreases in accuracy attributed to physiological arousal in high math anxious (HMA) individuals. Participants were instructed to use cognitive reappraisal (CR), expressive suppression (ES), or a “business as usual” strategy. During the ES condition, HMA individuals showed decreases in math accuracy associated with increased EDA, compared to low math anxious (LMA) individuals. For both HMA and LMA groups, CR reduced the association between physiological arousal and math accuracy, such that even elevated physiological arousal levels no longer had a negative association with math accuracy. These results show that CR provides a promising technique for ameliorating the negative relationship between math anxiety and math accuracy.

## Introduction

Math anxiety represents a significant challenge for many students, as anxious emotion creates obstacles for math achievement (Hembree, 1990). Math anxiety refers to the feelings of anxiety, nervousness, tension and apprehension related to anticipating or calculating mathematics (Hembree, 1990; Ma, 1999; Ashcraft, 2002; Suárez-Pellicioni et al., 2015; Namkung et al., 2019). In the present study, we explored the relationship between math anxiety, physiological arousal (measured by electrodermal activity, EDA) and math accuracy across various emotion regulation (ER) strategies. Our results suggest that especially compared to an ER strategy called expressive suppression (ES), cognitive reappraisal (CR) was associated with ameliorating the negative relationship between math anxiety, physiological arousal, and math accuracy.

### Math Anxiety

Math anxiety is thought to be related to other experiences of anxiety, such as general anxiety or test anxiety (Kazelskis, 1998; Kazelskis et al., 2000; Ashcraft, 2002; Suárez-Pellicioni et al., 2015; Dowker et al., 2016). However, math anxiety is associated with fairly specific negative emotions, thoughts, and performance deficits in mathematics; increased math anxiety is associated with deficits in math performance but not other difficult cognitive tasks or academic domains (Hopko et al., 2002; Pizzie and Kraemer, 2018; Pizzie et al., 2020a). High math anxious (HMA) individuals report increased negative experiences with mathematics, and math anxiety is also associated with decreases in math performance, with math anxious individuals showing decreased accuracy or proficiency in mathematics (Ma, 1999; Ramirez et al., 2013; Namkung et al., 2019). Math anxiety is also characterized by avoidance of mathematics, and math anxious individuals avoid even the mere presentation of mathematics (Pizzie and Kraemer, 2017), speeding through math at the expense of spending adequate time to solve the problem (Faust et al., 1996; Pizzie et al., 2020b). Over a longer timescale, math anxious individuals avoid taking further math classes, and avoid career choices that focus on math and quantitative skills (Betz, 1978; Hembree, 1990; Dowker et al., 2016).

Much of the previous research on math anxiety has focused on the cognitive components of math anxiety, exploring how math anxiety is related to math performance and cognitive processes that support math calculation (Ashcraft, 2002; Ashcraft and Moore, 2009; Moore and Ashcraft, 2013). Past research has established that working memory deficits accompany math anxiety and are related to deficits in math performance. Using the foundational literature on general anxiety as a point of comparison, the root of these working memory deficits are related to deficits in cognitive inhibitory mechanisms (Hopko et al., 1998). Increased math anxiety is associated with intrusive anxious thoughts that interrupt and distract verbal working memory, occupying working memory resources that would otherwise be devoted to mathematical calculations (Hopko et al., 1998; Beilock, 2008).

Interventions targeting math anxiety are largely categorized by two approaches: providing additional support for improved math understanding (i.e., intervening to provide additional practice in math calculation or improved math instruction), or intervening to ameliorate the feelings of anxiety (i.e., intervening at the level of emotion). Interventions targeting the quality or frequency of math instruction to improve the deficits associated with math anxiety have shown decreases in math anxiety and increases in math performance (Agarwal et al., 2014; Iuculano et al., 2015; Supekar et al., 2015; Pizzie and Kraemer, 2017). However, some of these interventions, such as one-on-one tutoring (Supekar et al., 2015), may be difficult to implement on a broader scale. Other research has identified anxious emotion as a focus for math anxiety intervention (Hembree, 1990; Jamieson et al., 2010, 2012, 2016; Ramirez and Beilock, 2011; Park et al., 2014). In the present study, we examine math anxiety from the perspective of affective science, and evaluate the introduction of emotion regulation strategies as a hypothesized intervention for math anxiety (Pizzie et al., 2020a), with this study focusing on the relationship between physiological arousal and task accuracy between HMA and LMA individuals.

In this psychophysiology experiment, we evaluated the effect of different emotion regulation (ER) techniques (Gross and Levenson, 1997; Gross, 1998; Gross and Thompson, 2007) on math performance in participants with high and low math anxiety, and explored the relationship between different regulation strategies with physiological arousal (electrodermal activity, EDA) and task performance. Specifically, we chose to focus on two emotion regulation strategies shown to be effective at reducing self-reported negative affect: cognitive reappraisal (CR) and expressive suppression (ES). We compared the effects of implementing these strategies across two different types of stimuli: math problems and analogies. Math problems were chosen as stimuli because they should be emotionally evocative for those who are HMA. Stimuli also included analogies, which can be considered a control condition. This analogy task was designed to be a working-memory intensive task that, although cognitively challenging, would not evoke the same negative emotions for HMA individuals.

### Physiological Arousal

In addition to negative thoughts and appraisals, anxiety is also associated with increased physiological arousal, here operationalized as electrodermal activity (EDA; Dawson et al., 2009; Figner and Murphy, 2011; Boucsein, 2012; Braithewaite et al., 2015). EDA is a correlate of sympathetic nervous system activity. Individuals experience increased activity in the sympathetic nervous system in varying degrees of readiness for a “fight, flight, or freeze” response, resulting increased physiological arousal across a number of different systems in the body (Dawson et al., 2009). In particular, researchers can measure this sympathetic nervous system activity by measuring the varying amount of microscopic sweat on an individual’s skin produced by eccrine sweat glands. The more sweat produced by these glands, the more electrical conductivity can be measured (Dawson et al., 2009; Figner and Murphy, 2011; Boucsein, 2012). As sympathetic nervous system arousal increases, we can measure increased EDA as a physiological correlate.

EDA is represented as a dynamic physiological response signal, and can be measured using the tonic skin conductance level (SCL; an average of a period of time), or specific skin conductance responses (SCR), representing specific phasic increases, usually in response to a specific stimulus (Nikula, 1991; Dawson et al., 2009; Figner and Murphy, 2011). However, either of these measurement approaches leaves something to be desired. SCL averages of a period of time, minimizing the peaks and valleys of the EDA signal. SCR measures the shape of the specific skin conductance response, measuring attributes of that response, such as the length and amplitude of the response to a stimulus. The responses that are characterized as SCRs do not always occur in response to every presentation of the stimulus, and so can create a selection bias in only analyzing these SCRs. For example, the amplitude of the SCR can only be measured for stimuli in which a SCR can be detected, which does not occur for every presentation of the stimuli. In this study, we have chosen to use an analysis technique that represents a compromise between these two approaches: calculating the area under the curve (AUC) of the electrodermal activity while participants are completing their calculations or estimations during the task (Figner and Murphy, 2011). Although this measure is more similar to analysis of SCL (i.e., averaging over a measurement of EDA over a specified period of time), this method also accounts for additional aspects of the EDA signal. In this way, we can calculate the level of activity during the entire response period, while also maintaining the curved shape and peaks associated with a SCR (Dawson et al., 2009; Bach et al., 2010; Boucsein, 2012).

Increased anxiety (Naveteur and Baque, 1987) and negative thought processes (Nikula, 1991) have been related to increased measurements of EDA. Math anxiety is associated with increased vigilance in response to mathematics, and previous research has indicated that biological correlates such as amygdala reactivity are associated with increased reactivity when HMA individuals are presented with mathematics (Young et al., 2012; Suárez-Pellicioni et al., 2013, 2015; Pizzie and Kraemer, 2017). However, past research has found mixed results when measuring physiological arousal through skin conductance. For example, past research reported no association between math anxiety and physiological arousal when measuring SCL over an extended period while individuals performed mathematics (Dew et al., 1984). More recent work suggests that physiological arousal is only associated with math anxiety when other cognitive factors are also taken into account (Strohmaier et al., 2020).

In the present study, we will examine the role of physiological arousal, measured by EDA, as a factor contributing to math accuracy. We will explore the idea that physiological arousal may be one of the factors that could distract or detract from math-related working memory processes within math anxiety. In other words, attention to physiological sensations and increased physiological arousal, and negative cognitive attributions of that physiological arousal, may be factors that contribute to the performance decrements observed in math anxiety (Blascovitch et al., 2003; Jamieson et al., 2012, 2016, 2020). In this way, our research will explore the relation between math anxiety, stimulus type, ER strategy, and physiological arousal, on task accuracy. This analysis will allow us to explore how math anxiety and ER strategy interact with physiological arousal and the relationship of these factors to math accuracy.

### Emotion Regulation Strategies

Cognitive reappraisal (CR) is the process by which individuals change their thought processes and appraisals about an emotional situation, thereby changing the meaning of the emotional stimulus and their physiological response (Gross, 1998). ES is an emotion regulation technique by which individuals suppress or hide their outward emotional response, keeping their feelings bottled up or repressed (Gross and Levenson, 1993). Foundational work using ER strategies such as CR illustrate the physiological benefits of utilizing these strategies to reduce negative affect (Gross and Levenson, 1997; Gross, 1998; Richards and Gross, 1999).

Emotional evaluation and regulation are dynamic processes, and the Extended Process Model details how ongoing feedback from emotion regulation has downstream consequences for emotional experience (Gross and Thompson, 2007). In the Extended Process Model, emotional experience is a dynamic and iterative process by which emotional cues lead to emotional response (Gross, 2008). That emotional response then creates feedback to inform the emotional situation and emotional cues (Ochsner and Gross, 2008; Gross, 1998, 2013). When we conceptualize an emotional situation that arises, this situation first includes emotional cues. Attention is first deployed to these cues and physiological sensations, then cognitive appraisals are made of the situation. These cognitive appraisals are key elements in which the individual interprets the cues and physiological sensations, finally leading to an emotional response. The biopsychosocial model posits that it is not necessarily the overall level of physiological arousal that determines the emotional experience, but instead it is the appraisal of that arousal that is key in understanding the relationship between arousal and cognitive performance (Blascovich, 1992). This resulting emotional response output then feeds back to inform the initial stages of the emotional evaluation, influencing the emotional cues.

When we consider how emotion regulation factors into this model, antecedent-focused coping focuses on changing the cues, thoughts and initial stages of the emotional experience, such as CR. CR transforms the meaning of the negative stimulus, changing the thoughts and appraisals and thereby down-regulating negative emotion in a way that affects both the initial appraisals of the stimulus as well as having more lasting effects (Jamieson et al., 2010; Denny and Ochsner, 2014). These approaches suggest that deficits in performance are not entirely about the level of physiological arousal *per se*, but rather the cognitive appraisal of the increased sympathetic nervous system activity that is associated with the task at hand (Blascovich, 1992). The cognitive appraisal of the emotional situation is determinant of the emotional response, such that increased physiological arousal may be appraised as a negative experience (i.e., “I’m nervous”) or a positive challenge (i.e., “I’m excited”). CR changes the cognitive appraisals of the situation, changing the emotional response and the interpretation of the emotional cues and physiological sensations. In this way, emotional cues and physiological arousal that would otherwise be interpreted as negative or stressful might be interpreted as an interesting challenge. Indeed, some previous research illustrates that CR can result in increased physiological arousal (measured by psychophysiological responses and fMRI) that in turn, is associated with *improved* task performance and *reduced* anxiety (Jamieson et al., 2010, 2012; McRae et al., 2010).

Further work examining CR in academic situations has found that CR of increased physiological arousal in stressful situations improves cardiovascular responses to stress (Jamieson et al., 2012), improves mathematics performance with both short- and long-term effects (Jamieson et al., 2010), and was associated with improved performance in a mathematics classroom environment (Jamieson et al., 2016). In a neuroimaging study, HMA individuals who were taught to use a CR strategy showed reduced negative ratings in response to math, increased accuracy in the math condition, and this increased accuracy was associated with increased neural activity in areas of the brain associated with arithmetic processing (Pizzie et al., 2020a).

In response-focused coping, such as expressive suppression (ES), the focus is on controlling the “end product” of the emotional experience, or controlling the emotional response (Ochsner and Gross, 2008; Gross, 1998, 2013). In the case of ES, although one may outwardly appear to have suppressed negative emotion, this ER strategy does nothing to change the internal experience of the negative emotion. In ES, negative cognitive appraisals associated with the stimulus are maintained, and result in increased vigilance and physiological arousal associated with maintaining a neutral outward appearance even though negative thoughts persist. ES may be especially taxing on working memory resources, because focusing on maintaining a neutral expression detracts from the working memory resources needed to devote to the task at hand. These instructed ER strategies (CR, ES) are often compared to a “business as usual” control condition, which we call “Look,” in which participants are instructed to respond to emotional stimuli as they normally would respond.

It is critical to evaluate not only how a particular intervention is associated with change in physiological arousal, but also how the intervention changes the association between arousal and task performance. In this study we examined how different regulation strategies and task contexts were associated with both arousal levels and task performance across HMA and LMA individuals.

### The Present Study

In this study, we evaluated how ER processes are associated with individual differences in math anxiety, hypothesizing that CR would be an effective technique for alleviating negative emotions as reflected in physiological arousal, and task performance. In contrast, we hypothesized that ES would not have a positive impact on task performance because this technique does not directly impact the negative cognitive appraisals when processing emotion. We hypothesized that because MA represents specific affective responses to mathematics, introducing ER strategies that might aid in regulating these responses would be associated with more advantageous responses to these math stimuli for those who are higher in MA. During this task, EDA was used as a measure of physiological arousal and measured during the presentation of the stimulus (i.e., math problems or analogies). We also hypothesized for HMA individuals, increased negative appraisals and distraction caused by physiological arousal would be related to decreased task accuracy. Individuals who report low levels of math anxiety (LMA) are not expected to show any difference between the math and analogy conditions, as they would not have the same negative cognitive appraisals of mathematics compared to HMA individuals. Although categories of stimuli were designed to be cognitively challenging, here we are comparing the specificity of the negative emotional responses associated with mathematics for HMA individuals.

For HMA individuals, if CR is related to the cognitive appraisals of the emotional scenario but is not directly related to the overall level of physiological arousal (such that even increased EDA may not be appraised as a negative emotional response), then CR would reduce the strength of the association between physiological arousal and task performance. This result would indicate that CR is associated with changing the negative appraisals associated when HMA individuals encounter math. In this case, these individuals may still experience increased physiological arousal, but this physiological arousal would no longer be appraised as negative, and would no longer be associated with decreased math accuracy. In contrast, we predict that ES, while reducing outward signs of anxiety, would not alleviate the negative cognitive appraisals of physiological arousal for HMA individuals solving math problems. However, by comparing ES and CR, we can compare two ER strategies that are both designed to reduce anxiety. Whether changing the appraisal of the anxiety (CR), or the outward appearance of anxiety (ES), we can determine whether these strategies differ in their ability to change the relationship between physiological arousal and math accuracy. Both of these instructed ER strategies will be compared to a “business as usual” strategy. In this way, we evaluated how math anxiety and ER strategies interact to be related to physiological arousal and task performance.

## Materials and Methods

### Participants

Undergraduate students were recruited for this experiment on the basis of their extreme scores on the Math Anxiety Rating Scale (MARS; Richardson and Suinn, 1972; Suinn and Winston, 2003). Students were recruited across multiple academic terms, and were recruited from a population enrolled in introductory psychology and neuroscience classes, who were offered course extra credit for completing a battery of questionnaires that included the MARS. Students were first recruited from an initial pool of 181 students, and this pool of participants was used to establish the range of math anxiety in this population (*M*_MARS_ = 2.24, *SD*_MARS_ = 0.40, *Range*_MARS_ = 1.44–3.6). Low math anxious (LMA, *Range* = 1.44–1.84) and high math anxious (HMA, *Range*_MARS_ = 2.66–3.6) students were selected to have scores that were approximately 1 SD below and above the mean of the sample, respectively. We chose to selectively recruit individuals from extreme ends of the scale to better highlight the differences between those who were high in anxiety, versus individuals who had relatively positive feelings toward mathematics. Ongoing recruitment for the subsequent terms used these score cutoffs (*LMA*_Max_ = 1.84, *HMA*_MIN_ = 2.66) as criteria to continue to recruit from a pool of an additional 307 students who were also enrolled in the subject pool (*Range*_MARS_ = 1.0–4.67).

From 58 students who participated in the study, three students were excluded from data analysis because they could not complete the task due to fatigue or power failure. One participant was excluded for having low overall accuracy (mean overall task accuracy = 53%, chance level responding = 50%), and two additional participants were excluded for a large number of missing responses (>3 SD above mean number of trials w/o a response, *M* = 3.8). The final sample for analysis included 52 participants (*M*_MARS_ = 2.26, *SD*_MARS_ = 0.81, *M*_*AGE*_ = 19.56, *SD*_*AGE*_ = 1.14, 63.5% female), 27 LMA participants (*M*_MARS_ = 1.53, *SD*_MARS_ = 0.22, *M*_*AGE*_ = 19.7, *SD*_*AGE*_ = 1.26, 44% female), and 25 HMA participants (*M*_MARS_ = 3.05, *SD*_MARS_ = 0.30, *M*_*AGE*_ = 19.4, *SD*_*AGE*_ = 1.0, 88% female). Although females (*M*_MARS_ = 2.51) and males (*M*_MARS_ = 1.84) significantly differ in self-reported math anxiety, *t*(44.34) = 3.27, *p* = 0.002, when gender was included in the subsequent analyses, it did not significantly interact with math anxiety. Therefore, although gender is an important consideration in math anxiety (Sokolowski et al., 2018), we did not find that gender was an important factor in consideration of these analyses, and will not discuss gender further in this manuscript.

Math anxiety is often confounded with trait anxiety (Hembree, 1990), therefore it is important to examine whether these findings are driven more by general anxious emotion or by math anxiety, *per se*. Consistent with this previously reported confound (Lyons and Beilock, 2012; Pizzie and Kraemer, 2017, 2018) scores on the MARS were significantly correlated with trait anxiety (STAI), *r*(*50*) = 0.55, *p* < 0.0001. To separately examine the effects of these two sources of anxiety, we calculated a measure of math anxiety controlling for trait (general) anxiety, by using residuals from a regression with the STAI-trait subscale (Spielberger, 2010) predicting MARS scores (Suinn and Winston, 2003; analyses conducted with raw MARS scores are reported in Supplementary Material). These residual scores were grouped such that scores above zero indicate that math anxiety was higher than what would be expected based on the relationship with trait anxiety, scores below zero indicating that math anxiety was lower. When participants were grouped on the basis of MA-STAI scores, six participants switched group membership. Three participants that were originally classified as HMA by their MARS scores alone were reclassified as lower anxiety when their high trait anxiety scores were taken into account (i.e., their reports of increased anxiety were not attributable to math alone), and three participants who were originally classified as LMA were classified as HMA. In the following analyses, we referred to these scores as MA groups indicating high (HMA) and low math anxiety (LMA).

Further descriptive statistics for these groups are also reported in Supplementary Table 4. All participants provided signed informed consent at the beginning of the study and were compensated with course credit or cash. All procedures in this experiment were approved by the Dartmouth College Committee for the Protection of Human Subjects.

### Task

#### Emotion Regulation Training

Modeled after traditional emotion regulation training paradigms (Gross and Levenson, 1997; Gross, 1998; Goldin et al., 2008; Ochsner and Gross, 2008; McRae et al., 2010; Silvers et al., 2012), participants were instructed that they would receive one of three instructions (“LOOK,” “SUPPRESS,” and “REAPPRAISE”) at the beginning of each block of trials (see Table 1), and that they would apply these techniques across the trials (20 trials) in a section of trials. Before participants began the trials, they were trained by the experimenter to use each ER strategy for each type of trials (see also Ochsner et al., 2012; Buhle et al., 2013). During the training, participants practiced using “LOOK,” “SUPPRESS,” and “REAPPRAISE” regulation techniques (Table 1).

**TABLE 1:** Emotion regulation conditions and stimuli.

**Condition:**	**“Business as Usual”**	**Cognitive Reappraisal (CR)**	**Expressive Suppression (ES)**
**Onscreen Cue:**	**LOOK**	**REAPPRAISE**	**SUPPRESS**
Excerpt from Task Instructions:	*“React as you normally would to each stimulus. While you may be conscious of your reactions, please do not try to change your response in such a way that would be different from your natural reaction.”*	*“Re-interpret the possible antecedents, outcomes and/or reality of the events you see in such a way that your emotional response is decreased. For example, you might reinterpret a math problem or analogy by imagining giving an explanation or teaching the problem to a friend. “*	*“Try to behave in such a way that a person watching you would not know that you were feeling anything. For example, to suppress any feelings you might have in response to a difficult math problem or analogy, you might try to control your tendency to look puzzled or appear frustrated.”*
Example of Application to Math Problems:	*“Went about it the normal way to decipher a math problem”*	*“Thought about teaching it to someone and how you would get to the correct answer”*	*“Got the answer wrong but tried not to show it on my face”*
Example of Application to Analogies:	*“Naturally thinking about the words, let [my]self react how I would normally if I were doing them for homework”*	*“Talked through each of the things I saw in my head; trying to think of how I would explain it to someone”*	*“Just tried to focus on being as stoic as possible”*

*Table comparing different instructed emotion regulation conditions with excerpts from the instructions given to participants as part of the training they received. Participants practiced all techniques with each task: math problems and analogies. In each of the examples, participants told the experimenter how they used the instructed technique in the example practice problems, and here we provide some illustrative examples of how participants explained how they implemented these techniques during the training.*

During the “business as usual” control condition (“LOOK”), participants were instructed to respond to the task using their normal, natural strategy, and that they should not try to change their reactions or expressions during these trials. They were instructed to respond to the stimuli however they normally would. The experimenter explained that during the blocks of ES trials (“SUPPRESS”), participants were asked to monitor and control their facial expressions to maintain a neutral expression, such that if they experienced any emotion, no one would know what they were feeling. During the CR training (“REAPPRAISE”), the experimenter explained CR as a method of viewing the stimuli in an objective manner (Ochsner and Gross, 2008; Denny and Ochsner, 2014). These instructions were in line with previous emotion regulation literature (Goldin et al., 2008; Silvers et al., 2012).

Because participants viewed various different kinds of stimuli, they were given examples for how to apply CR to pictures, math problems, and analogies. Here we focus on a type of CR known as self-distancing (Kross and Ayduk, 2011; Denny and Ochsner, 2014), encouraging participants to view the situation from a more objective perspective, thereby reducing negative affect. When participants were completing the math problems and analogies, participants were instructed to focus less on their own reactions to the problems and to instead concentrate on the steps of the problem, imagining that they were explaining the steps of solving the problem or completing the analogy to a friend. Or participants could imagine that their math teacher was the one explaining the solution to the problem. This distancing strategy was thought to target the ruminations that HMA individuals experience during math problems (Beilock, 2008), ameliorating these working memory deficits by encouraging individuals to focus on the steps of the problem using an internal narrative (Hopko et al., 1998; DeCaro et al., 2010; Shi and Liu, 2016).

The experimenter assessed whether the participant had effectively learned each technique by asking the participant to review their thought process during practice trials. The experimenter verbally assessed understanding after each block of trials. The experimenter redirected responses that were off-topic and helped to differentiate the techniques during practice trials (as needed) so that each participant understood how to apply each emotion regulation technique across the various kinds of stimuli. The experimenter verified that all participants had an effective understanding of the different regulation techniques by the end of the practice trials before continuing on to the task (see Table 1 for example responses). Examples of participant responses that needed to be redirected during the training usually included forgetting the task instructions (i.e., forgetting what it meant to “reappraise”), or needing to be redirected to focus on the important elements of the strategy. For example, if a participant remarked that they were “focusing on the elements of the [task],” or “trying to predict the answer” during the ES condition, the experimenter would redirect the participant to focus on maintaining an outwardly neutral expression or “poker face.” In the CR condition participants who needed to be redirected reported that “unsure what to reappraise since it’s not super emotional,” or that they were unsure as to whether they needed to control their outward expression of emotion, so the experimenter would redirect the participant to focus on viewing the problem objectively or explaining it to one’s friend. This training and set of practice trials took between 20 and 30 min to complete.

### Stimuli

Participants were told that they would apply these three strategies across different types of stimuli: math problems, analogies, and pictures (negative and neutral). Participants completed math problems, a task thought to be evocative of negative affect for HMA individuals, and analogies, a cognitively difficult comparison task that might not elicit negative affect in the same way for HMA individuals. Participants also completed a similar task with negative and neutral IAPS pictures (Lang and Bradley, 2007). These were categories of stimuli that were chosen to be similar to previous investigations of emotion regulation (McRae et al., 2010), and these stimuli are discussed in the Supplementary Material. These picture stimuli are further analyzed and discussed in Burr et al. (2021).

For the math trials, participants were presented with arithmetic problems on the stimulus screen [e.g., “(8 × 9) ÷ 3 × 9”], and asked to solve using order of operations. Participants indicated whether a value presented on the answer screen correctly solved the problem. Math problems were generated from a random problem generator for teachers (TheTeachersCorner.net worksheet generator). For analogies, participants first viewed an incomplete analogy (e.g., “DEFERENCE: RESPECT, affection:”), and were asked to decide if a word presented on the answer screen correctly completed the analogy (e.g., “love”), or incorrectly completed the analogy (e.g., “truth”). Analogies were drawn from previously published practice trials provided for the Graduate Record Exam. All participants completed the same set of all problems, and the order of these problems was randomly presented. Participants also made ratings of how negative and positive they felt, as well as how difficult they thought the trials to be; though due to data loss due and technical problems, these ratings will not be discussed.

**FIGURE 1:**
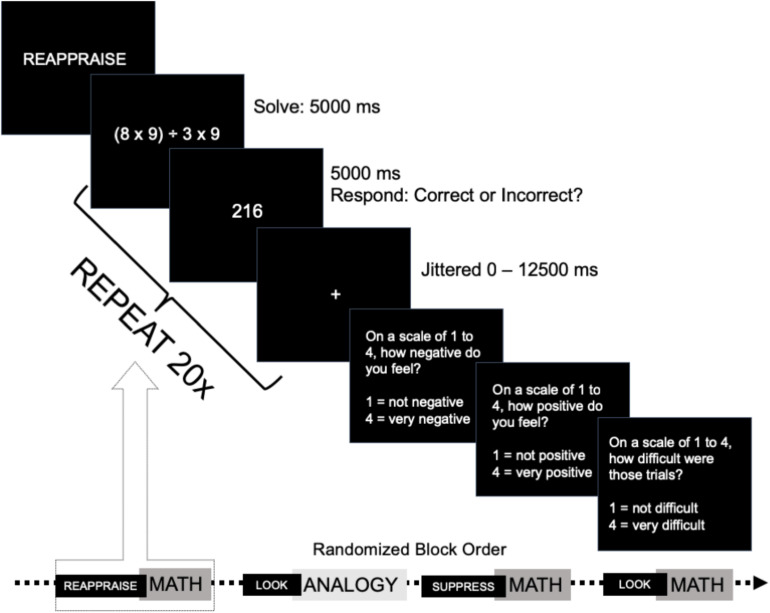
Trial cadence and sample block sequence for Emotion Regulation task. Sequence of events during trials for emotion regulation task depicting a math problem in the CR condition. Participants saw an emotion regulation instruction at the start of each block of trials. Then, participants completed a short sequence of trials (math trials pictured above). Participants completed 20 unique trials of a single type during each block of trials (trial procedure repeated 20×). At the end of each block of trials, participants were asked to indicate their negative and positive feelings, as well as to rate the difficulty of that block of trials.

Each block of 20 trials began with the ER strategy, and a cue that indicated the type of trials to be completed. For all trials, participants saw the initial stimulus (math problems, analogies, pictures) for 5,000 ms, and then were presented with an answer screen for 5,000 ms, on which participants were presented with a proposed solution to the problem. Participants were instructed to decide whether the answer presented on the screen was the correct solution to the problem, and press a button that recorded the accuracy and response time for that trial (correct/incorrect decision). The answer screen remained on the screen for the full 5,000 ms regardless of when the response was recorded. Each trial was followed with a jittered fixation (Figure 1).

Participants completed problems in all 12 categories of stimuli (3 (emotion regulation strategy: look, CR, ES) × 4 (stimuli: negative pictures, neutral pictures, analogy, math); see Table 1 and Figure 1). Participants completed 20 trials of each type across the three regulation conditions (20 “look”, 20 CR, 20 ES) such that participants completed 60 analogy trials and 60 math trials, as well as 60 negative picture trials, and 60 neutral picture trials, for a total of 240 trials across 12 conditions. All participants completed the same set of problems, and these were presented in a randomized order. The order of blocks was randomized for each participant to help reduce order effects, such that no participant received the same blocks or the same trials in each order. This randomization was designed such that our effects could not be attributed to holdover effects that would be consistent across conditions, and that within-subject comparisons of these conditions could not be attributed to consistent effects due to the previous block.

Each type of stimuli and ER strategy was compared in a within-subject design. In regards to the choice of a within-subject design versus a between-subject design, previous research has demonstrated that participants can switch back and forth between regulation techniques quite rapidly, even on a trial-by-trial basis (McRae et al., 2010). Here we chose to use a within-subject design, but one in which ER techniques varied not on each trial, but on each block of 20 trials. This block format allowed participants to effectively apply each technique across a section of trials (and for us to compare responses to different trial types within-subject). These blocks reduced distraction or increased working memory load that would have been created by constantly switching between ER strategies and cognitive tasks. Participants were given opportunities for self-timed breaks in between sections of 20 trials, which lasted a minimum of 10–15 s between blocks of trials.

### Questionnaires

Participants had previously completed the Math Anxiety Rating Scale (MARS; Richardson and Suinn, 1972; Suinn and Winston, 2003), a 30-item scale that assesses math anxiety at the beginning of the semester in which they completed the experiment. At the end of the experiment, participants also completed a series of questionnaires to determine their individual levels of anxiety across a number of different domains (Table 2), including trait anxiety (Spielberger State-Trait Anxiety Inventory—trait, STAI; Spielberger, 2010), test anxiety (Spielberger Test Anxiety Inventory, TAI; Spielberger, 2009), writing anxiety (WA; Daly and Wilson, 1983), ratings of positive and negative affect (PANAS; Watson et al., 1988), and habitual practices of emotion regulation using the ES and CR subscales of the Emotion Regulation Questionnaire (ERQ; Gross and John, 2003). Participants also provided demographic information.

**TABLE 2:** Demographic information and questionnaire scores.

**Measure [Mean (SD)]**	**All (*N* = 52)**	**LMA (*n* = 27)**	**HMA (*n* = 25)**
Age	19.56 (1.15)	19.63 (1.25)	19.48 (0.96)
Gender – % Female	63.6%	51.9%	76%
Math Anxiety Rating Scale (MARS)*	2.26 (0.81)	1.66 (0.50)	2.92 (0.52)
Math Anxiety Controlling for Trait Anxiety (MA-STAI)*	−0.003 (0.65)	−0.53 (0.36)	0.56 (0.44)
Spielberger Test Anxiety Inventory (TAI)*	1.91 (0.65)	1.67 (0.56)	2.16 (0.67)
Spielberger State-Trait Anxiety Inventory—Trait subscale (STAI)	2.06 (0.50)	1.96 (0.54)	2.17 (0.44)
Emotion Regulation Questionnaire- Cognitive Reappraisal subscale (ERQ—CR)	5.12 (0.90)	5.14 (0.88)	5.10 (0.93)
Emotion Regulation Questionnaire- Expressive Suppression subscale (ERQ—ES)*	2.87 (1.02)	3.23 (1.02)	2.48 (0.87)
Positive and Negative Affect Schedule—Negative Affect (PANAS—NA)	1.94 (0.70)	1.81 (0.70)	2.08 (0.69)
Positive and Negative Affect Schedule—Positive Affect (PANAS—PA)	3.45 (0.60)	3.51 (0.56)	3.39 (0.66)
Writing Anxiety (WA)	2.81 (0.67)	2.77 (0.68)	2.87 (0.68)

*Demographic and Questionnaire data presented for the current sample. LMA and HMA groups were formed on the basis of groups created by MARS scores controlling for STAI (MA-STAI). ^∗^Indicates that the HMA and LMA groups showed significant differences between groups on this measure, *p* < 0.05, where the HMA group had significantly higher scores on these variables than the LMA group.*

### Psychophysiology Data Collection

Electrodermal activity (EDA) was collected from the hand of each participant by attaching two Ag/Ag-Cl pre-gelled electrodes to the index and middle finger of the non-dominant hand (determined by asking participants which was their dominant hand, no participants reported being ambidextrous). Electrodes were placed over the second phalanx of each digit. EDA signals were submitted to a BioPac amplifier with a gain of 5 μΩ/V, and DC restored. EDA samples were collected at a rate of 1,000 Hz. To preprocess the data, we used a band pass filter between 5 and 60 Hz to isolate the signal of interest, and data were smoothed using mean value smoothing over a window of 500 ms (Figner and Murphy, 2011). We used responses collected during the 5,000 ms stimulus window and 5,000 ms response window. To account for the shape of the physiological response function over this time period, we calculated the integral, or area under the curve (AUC), of the skin conductance response separately during the stimulus and response periods. The EDA measure used for these analyses was the calculated AUC during the 5,000 ms stimulus period. To account for individual differences in baseline activity, all values were z-scored within each participant across all conditions.

## Results

### Overview

Our main hypothesis concerns the comparison between the three emotion regulation strategies (CR, ES, and “look”) on the effect of reducing the strength of relationship between physiological arousal and performance for HMA participants compared to LMA participants, evaluating accuracy as an outcome measure. The test of this hypothesis is therefore a comparison of two effects within a four-way interaction in which task performance is predicted by the interaction of ER strategy (3: CR, ES, look) by math anxiety group (2: HMA, LMA) by stimulus type (2: math, analogy) by physiological arousal (EDA), as shown in Figure 3.

**FIGURE 2:**
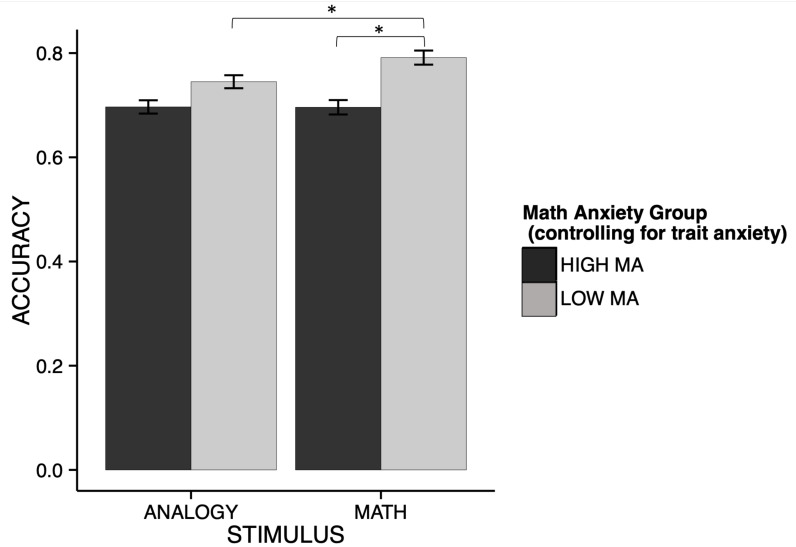
Interaction between math anxiety and stimulus type for accuracy. The pattern of accuracy across stimulus type differs on the basis of math anxiety. Overall, the LMA group shows higher accuracy than the HMA group [*χ^2^*(1) = 13.83, *p* < 0.001], however, this performance gap between groups interacts significantly with stimulus type [*χ^2^*(1) = 4.03, *p* = 0.045]. For the analogy condition, the LMA group slightly outperforms the HMA group. This disadvantage for high math anxiety is even greater in the math condition, indicating that math anxiety creates an additional performance decrement in the math condition for HMA individuals. *indicates pairwise comparisons that are statistically significantly different at *p* < 0.05.

**FIGURE 3:**
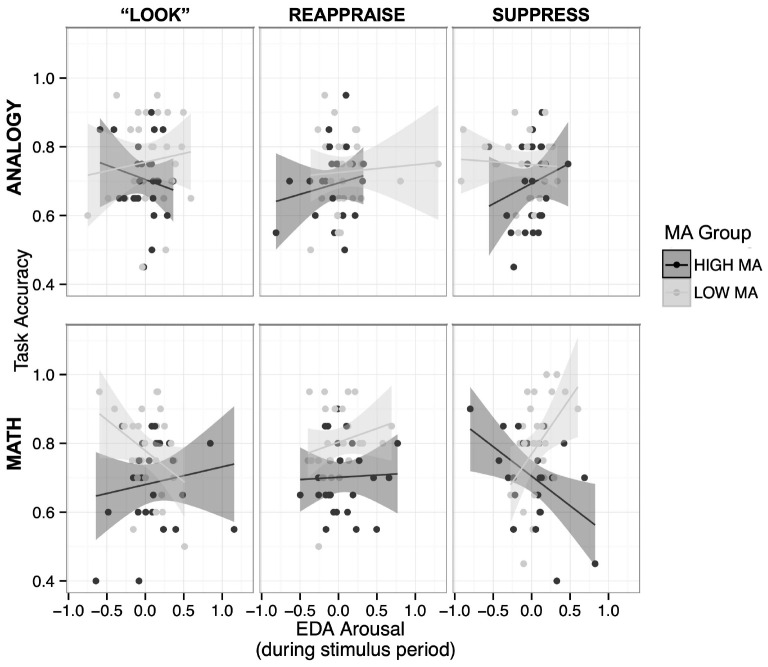
Accuracy scores determined by 4-way interaction between MA group, stimulus type, ER strategy, and physiological arousal (EDA). When examining the effects of math anxiety, EDA, task, and ER strategy on task accuracy, we find a 4-way interaction, *χ^2^*(2) = 15.69, *p* = 0.0003. Whereas HMA and LMA groups differ significantly across “Look” and ES instructions, CR reduces the association between arousal on math performance for both groups, indicating that CR is an effective strategy for HMA individuals to reduce the harmful effects of anxiety on math performance.

Analyses were conducted as follows using R (R Development Core Team, 2008) with RStudio. We computed linear mixed models (LMMs) with the lme4 (Bates et al., 2015) and lmtest packages in order to estimate statistical significance of fixed effects within these models (Zeileis and Hothorn, 2002). Pairwise comparisons were performed with the package *emmeans* (Lenth, 2021). Plots and graphics were made using ggplot2 (Wickham, 2009). In order to estimate the effects of our LMMs, we constructed this model with our fixed factors math anxiety (between subject fixed effects) and additional experimental variables (stimulus type, and emotion regulation strategy; all within-subject factors), and random effects accounting for individual differences among participants. We chose to utilize LMMs as we felt this analysis technique to comprehensively analyze the categorical and continuous predictors in our analyses. We prioritized analyses comparing across levels of math anxiety as primary analyses, and analyses with other separate aspects of anxiety (e.g., test anxiety) as further exploratory analyses, which are not reported in this manuscript (see Supplementary Material).

Estimates of statistical power for each of the analyses presented in the manuscript were estimated using the *mixedpower* package in R (Kumle et al., 2020)^1^. This package uses a simulation based on the existing data set to determine estimated statistical power (the proportion of statistically significant models out of the total number of simulated models) on the basis of a specific model at varying simulated sample sizes. In order to explore the statistical power associated with the analyses reported here, please see the Supplementary Material and Supplementary Tables 1–3 for further explanation of the estimated statistical power associated with the analyses in this manuscript.

### Behavioral Results

#### Accuracy: Stimulus Type × ER Strategy × MA

We calculated an LMM evaluating the effect of math anxiety (fixed effect: HMA, LMA), stimulus type (fixed effect, stimuli: analogy, math), and emotion regulation (fixed effect, emotion regulation: look, CR, ES), on task accuracy was calculated across all participants (random effects accounting for individual differences across participants; REML criterion at convergence, −406.5). Using this model, we found no main effect of stimulus type *χ^2^*(1) = 0.02, *p* = 0.97, no main effect of ER strategy, *χ^2^*(2) = 0.003, *p* = 0.99. We found a significant main effect of MA group, *χ^2^*(1) = 13.83, *p* = 0.0002. Low MA participants (*M* = 0.77, *SE* = 0.01) scored significantly higher than high MA participants (*M* = 0.70, *SE* = 0.01), *t*(50) = −3.72, *p* = 0.0005. We found an interaction between math anxiety group and stimulus type *χ^2^*(1) = 4.03, *p* = 0.045, such that LMA individuals show a performance advantage across both stimulus types, but this is exaggerated in the math condition (Figure 2). *Post hoc* t-tests were conducted to understand the effects within this interaction. Within the math condition, HMA individuals (*M* = 0.70, *SE* = 0.02) scored significantly lower than LMA individuals (*M* = 0.79, *SE* = 0.02), *t*(90.8) = −4.22, *p* = 0.0001. Within the analogy condition, HMA individuals (*M* = 0.70, *SE* = 0.02) scored significantly lower than LMA individuals (*M* = 0.75, *SE* = 0.02), *t*(90.8) = −2.14, *p* = 0.035. Whereas LMA individuals showed significantly increased accuracy in the math condition compared to the analogy condition, *t*(250) = −2.86, *p* = 0.005, HMA individuals did not show significant differences in accuracy between stimuli, *t*(250) = 0.040, *p* = 0.97. This result replicates previous findings with regard to math anxiety, such that more math anxious individuals tend to show exaggerated deficits with regard to mathematics, even compared to other difficult cognitive tasks (Ashcraft, 2002; Suárez-Pellicioni et al., 2015; Pizzie et al., 2020a,b). Here we find that this effect is right at the threshold of significance, and may be slightly underpowered (see Supplementary Material) and so should be interpreted with caution. However, we chose to include some discussion of this effect, especially as it relates to previous literature demonstrating these domain-specific deficits for individuals with increased math anxiety.

There was no interaction between stimulus type and ER strategy, *χ^2^*(2) = 0.67, *p* = 0.71, no interaction between ER strategy and MA group, *χ^2^*(2) = 0.1, *p* = 0.95, and no three-way interaction between stimulus type, ER strategy, and MA group, *χ^2^*(2) = 0.27, *p* = 0.87. Thus, using a measure of math anxiety that eliminated the association of broader patterns of negative affect (trait anxiety), we replicated previous findings that show that MA is associated with specific deficits in math performance. Neither the distancing CR instructions nor the ES instructions resulted in a direct improvement in task accuracy.

When evaluating reaction time, we find significant differences associated with stimulus type, but all other main effects and interactions were not statistically significant effects. Here our hypotheses focus on accuracy as an outcome measure, and the relationship between math anxiety, task performance, ER strategies, and in our next analysis, physiological arousal. For results related to reaction time, please see Supplementary Material.

### Psychophysiological Results

#### Accuracy: Stimulus Type × **ER Strategy** × **EDA** × **MA**

In our final set of analyses, we used EDA as a predictor variable as we investigated the relationship between physiological arousal and math and analogy accuracy, in addition to our other factors of math anxiety, stimulus type, and ER strategy. This set of analyses comprises the most direct test of our main hypotheses—here we examine whether the two HMA and LMA groups show different relationships from one another across different ER strategies.

We constructed an LMM to predict accuracy with stimulus type, ER strategy, EDA, and MA group as fixed factors, with random effects for each participant (REML criterion at convergence: −373.6). Importantly, and consistent with our main hypotheses, we found a significant four-way interaction between MA group, stimulus type, ER strategy, and EDA, *χ^2^*(2) = 15.69, *p* = 0.0003 (Figure 3). The main focus of this analysis is on accuracy in the math conditions, examining the relationship between EDA and accuracy across the ER strategy conditions for each of the math anxiety groups.

We utilized *post hoc* t-tests to further explore these complex relationships, focusing on the math condition (Table 3 and Figure 3). First, within each ER strategy, we calculated differences between MA groups. This analysis allows us to compare the differences between each group for the relationship between EDA and math accuracy within each ER strategy. In the ES condition, we find that the relationship between EDA and accuracy is different between the HMA and LMA groups, *t*(270) = −3.404, *p* = 0.0008. In the math ES condition, HMA individuals show a negative relationship between EDA and math accuracy (β_HMA–Math–__ES_ = −0.12), whereas LMA individuals show a positive relationship between EDA and math accuracy (β_LMA–Math–__ES_ = 0.31). In the math “Look” condition, we again find that the relationships between EDA and math accuracy significantly differ between HMA and LMA groups, *t*(269) = 2.09, *p* = 0.038. In the math “Look” condition, HMA individuals (β_HMA–Math–Look_ = 0.036) have a neutral or slightly positive relationship between EDA and accuracy, and LMA individuals (β_LMA–Math–Look_ = −0.18) show a negative relationship between EDA and accuracy. However, in the math CR condition, the slopes do not significantly differ between groups, *t*(270) = −0.673, *p* = 0.50. In the math CR condition, HMA individuals (β_HMA–Math–CR_ = −0.02) and LMA individuals (β_LMA–Math–CR_ = 0.06) both show a relatively neutral relationship between EDA and math accuracy. Despite significant variation between groups in the ES and “Look” strategies, this difference in slope between groups is ameliorated in the CR condition. In the CR strategy, we do not observe a strong relationship between EDA and math accuracy, and the HMA and LMA groups no longer differ significantly, meaning that the amount of arousal experienced by these individuals is not strongly related to math task performance for either group.

**TABLE 3:** Pairwise comparisons in interaction between EDA, MA group, ER strategy, and stimulus type, with accuracy as an outcome measure.

**Interaction: EDA × MA group × ER strategy × Stimulus type with accuracy**			
**Condition**		**Group comparison β values**	**Pairwise comparison**
**Comparing MA groups**			
	Math-ES	HMA: β = −0.12 LMA: β = 0.31	*t*(270) = −3.404, *p* = 0.0008**
	Math-Look	HMA: β = 0.036 LMA: β = −0.18	*t*(269) = 2.09, *p* = 0.038*
	Math-CR	HMA: β = −0.02 LMA: β = 0.06	*t*(270) = −0.673, *p* = 0.50

**Comparing ER strategies**			
	HMA	Look: β = 0.036 CR: β = −0.02	*t*(272) = 0.54, *p* = 0.92
	HMA	Look: β = 0.036 ES: β = −0.12	*t*(265) = 1.85, *p* = 0.19
	HMA	CR: β = −0.02 ES: β = −0.12	*t*(272) = 1.10, *p* = 0.62
	LMA	Look: β = −0.18 CR: β = 0.06	*t*(275) = −2.04, *p* = 0.12
	LMA	Look: β = −0.18 ES: β = 0.31	*t*(274) = −3.49, *p* = 0.0017**
	LMA	CR: β = 0.06 ES: β = 0.31	*t*(261) = −1.94, *p* = 0.15

This table depicts pairwise comparisons within the math condition in the four-way interaction with the factors EDA, MA group, ER Strategy and Stimulus type, with accuracy as an outcome measure. The table focuses on pairwise comparisons within the math condition only. Pairwise comparisons were made evaluating the differences between groups within each strategy. Additionally, within each group, pairwise comparisons were computed between each of the ER strategies. *indicates pairwise comparisons that are statistically significantly different, *p* < 0.05. **indicates pairwise comparisons that are statistically significantly different, *p* < 0.01.

We also wanted to know whether individuals within each MA group showed differences the association between EDA and accuracy comparing between ER strategies (Table 3 and Figure 3). These comparisons would allow us focus on each group and variation associated with the ER strategies. In the HMA group, the relationship between EDA and accuracy did not significantly vary when comparing “Look” and CR, *t*(272) = 0.54, *p* = 0.92, “Look” and ES, *t*(265) = 1.85, *p* = 0.19, and CR and ES, *t*(272) = 1.10, *p* = 0.62. Within the LMA group, the relationship between EDA and accuracy did not significantly vary when comparing “Look” and CR, *t*(275) = −2.04, *p* = 0.12, and CR and ES, *t*(261) = −1.94, *p* = 0.15. Within the LMA group in the math condition, the relationship between EDA and accuracy significantly varies between the “Look” and the ES strategies, *t*(274) = −3.49, *p* = 0.0017. In comparing between the ER strategies, we do not find that HMA individuals had significant variation in EDA and accuracy across ER strategies within the math condition. We do find that within the LMA group, there was significant variation in the relationship between EDA and accuracy, especially when comparing the “Look” (β_LMA–Math–Look_ = −0.18) and ES (β_LMA–Math–ES_ = 0.31) conditions. We do not find that any of the group comparisons or comparisons between ER strategies for the analogy condition result in significant differences in the relationship between EDA and analogy accuracy, all *p*’s > 0.20.

*Other comparisons and interactions.* There was a main effect of MA group on accuracy, *χ^2^*(1) = 12.27, *p* = 0.0004 (see previous accuracy section for description). There was no main effect of stimulus type on accuracy, *χ^2^*(1) = 0.02, *p* = 0.89, no main effect of ER strategy, *χ^2^*(2) = 0.11, *p* = 0.95, and no main effect of EDA on accuracy, *χ^2^*(1) = 0.17, *p* = 0.68. There was no significant interaction between stimulus type and MA group on accuracy (discussed previously), *χ^2^*(1) = 3.07, *p* = 0.08. There was no two-way interaction between stimulus type and ER strategy on accuracy, *χ^2^*(2) = 0.73, *p* = 0.69, no interaction between stimulus type and EDA, *χ^2^*(1) = 2.09, *p* = 0.15, no interaction between ER strategy and EDA on accuracy, *χ^2^*(2) = 0.75, *p* = 0.69, there was no two-way interaction between ER strategy and MA group on accuracy, *χ^2^*(2) = 0.17, *p* = 0.92, and no interaction between EDA and MA group on accuracy, *χ^2^*(1) = 0.22, *p* = 0.63. There was no significant three-way interaction between stimulus type, ER strategy, and EDA, *χ^2^*(2) = 5.52, *p* = 0.06. There was no three-way interaction between stimulus type, ER strategy and MA group on accuracy, *χ^2^*(2) = 1.02, *p* = 0.60, there was no three-way interaction between stimulus type, EDA, and MA group on accuracy, *χ^2^*(1) = 2.51, *p* = 0.11, and no three-way interaction between ER method, EDA, and MA group on accuracy, *χ^2^*(2) = 2.72, *p* = 0.26.

In an additional analysis, we also compared how the addition of physiological arousal (EDA) to the model predicting accuracy affects the total amount of variance accounted for within accuracy. In this way, we can gain a better understanding of whether accounting for EDA as an additional factor is meaningful in understanding task accuracy. We used the anova() function in R to evaluate the whether the variance accounted for by the addition of math anxiety represents a measurable improvement in the model (using maximum likelihood estimation). In this analysis, we compared the variance accounted for by the LMM model of accuracy with MA group + stimulus type + ER strategy to an identical model with the addition of EDA. In comparing these two models, we find that the model accounting for EDA accounts for significant additional variance, *χ^2^*(12) = 22.96, *p* = 0.028.

Additional analyses evaluating EDA as an outcome measure are included in the Supplementary Material. We did find a significant three-way interaction between math anxiety, stimuli, and ER strategy. However, in exploring the pairwise comparisons of this analysis, we did not find that planned comparisons revealed significant differences that were relevant to our hypotheses, and these analyses are discussed further in Supplementary Material.

*Summary.* Whereas we observe group differences between HMA and LMA individuals in the ES and Look conditions, when using the CR strategy, the association between EDA and performance is no longer a strong relationship for either the HMA or LMA groups. It is especially interesting that this CR strategy seems to reduce the association between physiological arousal and performance for both the HMA and LMA individuals, such that neither group differs in the association between physiological arousal and performance in math. The lack of association between EDA and accuracy that we observe in the CR condition for math for both MA groups seems to mirror the lack of strong association that we observe for the analogy condition. Because the analogy condition doesn’t have the same affective loading as math, we do not observe a strong relationship between physiological arousal and accuracy in the analogy condition, and we see a similar lack of association in the math CR condition. These results suggest that CR is a cognitive strategy for both HMA and LMA individuals to reduce the association between negative affect and physiological arousal on performance. These effects are subtle, and should be interpreted with caution, but provide a promising starting point with regard to understanding the relationship between math anxiety and ER strategies.

## Discussion

In this experiment, we examined the association between emotion regulation and math anxiety. We explored the relationship between math anxiety and math accuracy, compared to a similarly difficult cognitive task (analogies), and how this association varied between math anxiety groups and ER strategies. We also explored how physiological arousal, as measured by EDA, was related to task performance. The results of this study are subtle, with our main results suggesting that CR as an ER strategy disrupts the relationship between appraisals of an emotional experience, physiological arousal, and task accuracy. Importantly, the results of this study suggest that CR mitigates the group variation between physiological arousal and accuracy for both LMA and HMA groups. Despite showing group differences in the ES and “Look” conditions, CR was associated with minimizing group differences between HMA and LMA individuals, with both groups showing little association between physiological arousal and math accuracy.

In contrast, during the ES condition, HMA and LMA individuals show differing relationships between physiological arousal and task accuracy. HMA individuals showed a decrease in accuracy associated with increased EDA in the ES condition (i.e., when ES fails to reduce physiological arousal, performance has a negative association between physiological arousal and performance). We observed a significantly different relationship between EDA and accuracy for LMA individuals. In the math ES condition, for HMA individuals, we hypothesize that increased physiological arousal was representative of ER failure—attempting to control negative affect by suppressing those feelings, and failing, resulting in an ironic rebound in EDA (Gross and Levenson, 1993). The ES instructions only concern outward appearance of emotion and not changing the underlying thought process, and do not change the cognitive appraisals that may continue to interpret the emotional cues and EDA as negative. Our finding that the ES condition is associated with a negative relationship between EDA and accuracy should be indicative of the fact that negative appraisals maintained during ES interfered with performance for HMA individuals, perhaps through increased working memory load. Consistent with this finding, previous research on ES has suggested that ES is associated with performance deficits and decreased activity in brain regions that support memory and cognition (Richards and Gross, 1999; Binder et al., 2012). Therefore, CR showed an advantage over ES because it reduces the association between group differences in physiological arousal and performance: both HMA and LMA groups did not differ in the relationship between EDA and math task accuracy.

HMA and LMA individuals show significant differences in the relationship between EDA and accuracy for both ES and the “Look” condition, however, these variations between groups are ameliorated in the CR condition. Both HMA and LMA groups showed no strong relationship between increased physiological arousal and math task accuracy in the math CR condition. Though both groups may have experienced increased physiological arousal in the math task, CR eliminated the group differences in the relation between increased physiological arousal and performance. In our CR instructions, we encouraged participants to imagine a low-pressure context for doing mathematics: explaining the problem to a friend, or imagining that they might take the place of their math teacher, instructing others using their expert knowledge. Using distancing (Gross and Levenson, 1993, 1997; Denny and Ochsner, 2014) allowed participants to again focus on the steps of completing the math problem. By utilizing this strategy, participants were encouraged to change their cognitive appraisal of the emotional cues and physiological arousal to be less negative, changing the emotional response.

Interestingly, the math CR condition appears to have similar results to those observed in the analogy condition, wherein participants did not show a strong association between physiological arousal and performance, likely because the analogy condition is devoid of negative emotional appraisals. Further, these results indicated that using CR through distancing allowed both LMA and HMA individuals to successfully regulate physiological arousal and cognitive appraisals, and encouraged success in mathematical problem solving regardless of increased physiological arousal.

This technique encouraged success even in the face of increased physiological arousal, providing a complement to other CR techniques, such as reframing, which are the preferred techniques used in other studies that focus on cognitive or academic tasks (Jamieson et al., 2010, 2012, 2016). This research is also consistent with additional neuroimaging research that suggests that CR is associated with decreased negative affect for HMA individuals, increased accuracy, and this increase in accuracy was associated with increased activity in areas of the brain that subserve mathematical calculation (Pizzie et al., 2020a). In this way, our results are supported by previous research that emphasized the importance of cognitive appraisals in resulting cognitive performance.

### Limitations

This study is met with some limitations. Most importantly, our research examines individual differences in math anxiety, and therefore, any results that we observe are associative in nature and cannot reflect causal inferences. The results presented in this study are subtle, and we look to future research to continue to bolster the hypotheses and conclusions drawn from this study. In this study, we have explored how these individual differences are associated with differences in behavioral and physiological factors, but further research should explore some of the underlying causal relationships with respect to math anxiety.

An additional limitation to consider is that although we originally selected the math and analogy tasks to be roughly equivalent in difficulty, in this sample of participants, analogy was the more difficult task overall. However, even though individuals showed higher accuracy in the math task overall, we did observe differences related to math anxiety in this task that are not present in the analogy task. Thus, although the math task proved to be the less difficult task overall, accuracy interacted with math anxiety across stimulus types; specifically, math anxiety was determinant of accuracy in mathematics compared to a more difficult, non-mathematical task.

An additional limitation arises from the range of math anxious individuals recruited for this study. We originally chose to recruit groups of participants based on their particularly high or low scores on the MARS, removing the mid-range of math anxiety. While this decision was necessary to highlight the differences between LMA and HMA individuals, we are hesitant to make conclusions about the ways in which moderate math anxiety may be related to performance. The present study was limited by the exclusion of this moderate population, and future research would be strengthened by examining the full range of math anxiety. Although we selected undergraduate students who scored at extremes of the range of our measure of math anxiety, the students in our population represent only one example of how students experience math anxiety, and show decrements in performance related to math anxiety. In addition, in controlling for trait anxiety, a small number of participants “switched” group membership. It is essential to control for variance associated with trait anxiety (Pizzie and Kraemer, 2018), however, further research should continue to consider how we draw distinctions between “higher” and “lower” math anxiety. The students in our sample represent highly competitive and high-achieving students. Conducting this experiment with different population of students, for example, students in a remedial math course at a community college, as in Jamieson et al. (2016) may have yielded different results as these students may show more extreme decrements to math performance than our sample. We hope that future research will continue to explore how different demographics and academic contexts are related to the experience of math anxiety.

Further, although our study utilized a form of CR focused on distancing, which was effective in reducing the relationship between EDA and accuracy, previous research has emphasized that reframing, or changing the meaning of a negative emotional response to focus on effectively dealing with challenges, has also been effective in improving math performance (Jamieson et al., 2010, 2012, 2016; Pizzie et al., 2020a). While we hypothesize that utilizing the reframing technique would be advantageous for HMA individuals, we cannot determine if, or how, different CR techniques would be associated with physiological arousal and performance across HMA and LMA individuals. Future research should explore how utilizing different CR techniques might be related to math outcomes, with the expectation that individuals may gravitate toward different emotion regulation techniques based on their own emotion regulation habits (Mauss et al., 2007). Though we cannot compare these techniques in the present study, we find it promising that both distancing and reframing have shown positive effects on mathematical performance, especially for individuals experiencing increased anxiety. Indeed, given the promising longitudinal results demonstrated by previous research on CR, and our results suggest that CR may be a positive strategy for math anxious individuals to employ, future research should continue to explore the longitudinal effects of CR, especially as it relates to real-world educational outcomes.

## Conclusion

The goal of this study was to examine the use of different emotion regulation strategies as a way to remediate the negative association of anxiety with math performance in math anxiety. Our results suggest that CR—encouraging participants to use a distancing strategy to think about explaining the steps of the problem in a low-anxiety scenario—was effective in reducing the association between physiological arousal and math task accuracy. Without providing any further instruction in math, CR allowed HMA and LMA individuals to focus on the steps of the problem at hand, effectively regulating physiological arousal and eliminating the relationship between increased physiological arousal and accuracy. These results provide an interesting starting point for additional research to continue to explore the relationships between cognitive appraisals, physiological arousal, math anxiety, and math performance. Regulating math anxiety by using CR encouraged HMA individuals to ameliorate the relationship between physiological arousal and accuracy, and allowed them to focus on successfully completing mathematics unburdened by the negative implications of math anxiety.

## Data Availability Statement

The raw data supporting the conclusions of this article will be made available by the authors, without undue reservation.

## Ethics Statement

The studies involving human participants were reviewed and approved by Dartmouth Committee for the Projection of Human Subjects. The patients/participants provided their written informed consent to participate in this study.

## Author Contributions

RP and DK both collaborated on the study ideas and design, wrote the manuscript. RP was responsible for data collection and analysis, with the oversight of DK. Both authors contributed to the article and approved the submitted version.

## Conflict of Interest

The authors declare that the research was conducted in the absence of any commercial or financial relationships that could be construed as a potential conflict of interest.

## Footnotes

1 https://psyarxiv.com/vxfbh/
