# Supplementary material for: The Association Between Emotion Regulation, Physiological Arousal, and Performance in Math Anxiety
Source: Front Psychol. 2021 May 11;12:639448. doi: 10.3389/fpsyg.2021.639448 (PMC8144633; doi:10.3389/fpsyg.2021.639448)
Supplement: Supplementary file 1 [file Data_Sheet_1.docx]

SUPPLEMENTARY MATERIAL

Method

**Power Analysis**

**Power simulation**

Our original power analysis was completed before data collection began in 2015, and as a result, reflects some of the methodology of the time. In the intervening time, new methods have been developed for analysis and for estimating power of various analyses. In this manuscript, we utilized linear mixed effects models to understand the relationship between math anxiety, stimulus type, emotion regulation strategy, and physiological arousal. As our sample size is potentially a bit small to estimate the effects of interest for these interactions, we decided to use a simulation in order to calculate estimated statistical power based on varying sample sizes using a packaged called mixedpower (Kumle, Vö, Draschkow, 2020, preprint available: <https://psyarxiv.com/vxfbh/>). This package uses successive iterations of the data to estimate power of the specified fixed effects with various plausible sample sizes. This package estimates statistical power by calculating the proportion of simulations in which the effect was significant compared to the total number of simulations.

In the following tables (Supplementary Table 1-3), we will report the estimated power for each of the analyses included in the paper at various sample sizes. For the model of accuracy with fixed factors of stimulus type, ER strategy, MA groups and EDA (with random effects for participants), for a sample size of 50-60 participants the 4-way interaction between these fixed factors has between .68-90 statistical power (computed as the proportion of statistically significant simulations out of the total with that simulated sample size). For example, one effect that we discuss, the interaction between math anxiety groups and stimulus type for accuracy is perhaps a bit underpowered (estimated power .48-.56). However, we include and interpret this effect with caution because this result also replicates previous research that demonstrates that the deficits associated with increased math anxiety are associated with decreases in math performance, but not necessarily with other difficult cognitive tasks (Ashcraft, 2002; Pizzie, Raman & Kraemer, 2020; Pizzie, McDermott, Salem & Kraemer, 2019; Suarez-Pellicioni, et al., 2015). We include this effect with respect to the broader literature on math anxiety, but because this effect is marginally statistically significant, and is slightly underpowered, we would encourage further research on this topic to continue to investigate these anxiety-related deficits. We hope that readers may consider the statistical power of the effects of interest when reviewing the results in the paper (statistically significant effects from the manuscript are indicated on in Supplementary Table 1-3).

Original Power analysis:

To compute a-priori sample size in order to have adequate statistical power for this experiment, we used G*Power 3.1 to estimate sample size from an effect size of from a study on emotion regulation techniques. We utilized a previously published study {Mella:2011jo} that also used skin conductance responses as the outcome measure of interest, and manipulated cognitive focus and intensity of the emotional stimuli presented (sounds). We utilized the effect size from a two-way interaction comparing cognitive focus (3 levels) x emotional intensity (3 levels) in a within-subject ANOVA, with skin conductance as the outcome. This interaction shows a moderate effect size for this within-subjects comparison, *η_p_^2^*= .14, *f*^2^ = .40 (Cohen a suggests *η_p_^2^* = 13 and Cohen’s *f^2^* =.3-.5 is a moderate effect size (Cohen, 1977; Mella, Conty, & Pouthas, 2011). We estimated the number of subjects based on an idealized analysis using a mixed ANOVA procedure (our hypothesized method of analysis at the time of this *a priori* analysis), with 2 between-subjects groups (HMA and LMA), and two within-subjects factors (3 emotion regulation strategies x 4 stimulus types; 12 measurements in all). Estimating power for the within-subjects analysis using and effect size of *f*^2^ = .40, a two-tailed alpha of .05 and 80% power, we would need at least 6 total participants to estimate the within-subject effects. For the between subject effects with the same parameters (2 groups, 12 measurements, 80% power, alpha of .05, *f*^2^ = .40 and correlation between measures = .6), we would need 34 total participants to estimate the between-groups effects. For the between-within interaction with these parameters, we estimate we would need a minimum of 6 subjects total. We recruited 58 students for this experiment. These power analyses were completed utilizing procedures and effect sizes before the year 2015, when power analysis procedures began to change. Although this sample size seems small, we recognize that it represents a minimum sample size, and that the individualized nature of the analysis that were computed in this manuscript may be slightly statistically underpowered. We recognize this as a potential limitation of the study, and we encourage future research to continue to examine these experimental questions to shed light on the relationship between emotion regulation and math anxiety.

**Stimuli**

The negative and neutral trials were not discussed in the manuscript, but we provide this methodological data here: Negative and Neutral images were drawn from the International Affective Picture System (IAPS; Lang, Bradley, & Cuthbert, 2008), and were selected to be negative in valence and high in arousal for the negative pictures (negative pictures; M_valence_ = 1.74, SD_valence_ = 0.17, M_arousal_ = 6.37, SD_arousal_ = 0.58), and neutral in valence and low in arousal for the neutral images (neutral pictures; M_valence_ = 5.06, SD valence = 0.10, M arousal = 3.07, SD_arousal_ = 0.50). For both neutral and negative picture trials, participants were shown an image on the stimulus screen and the answer screen. During the answer screen, participants were asked to indicate whether the second image presented was identical to the previous image (a “correct” trial), or whether the image had been slightly altered using Photoshop (an “incorrect” trial).

When participants were instructed to use reappraisal, participants were instructed to use self-distancing when responding to the photographic stimuli. For example, participants were instructed to view the pictures by focusing on the details of the photograph, and imagining that they were photographer or medical professional, viewing the photograph stimuli from a professional perspective (self-distancing), or generating a personal narrative that made the stimulus appear less negative by focusing on details of the photograph (e.g., “Although at first I thought he looked lonely and sad, I imagined the man pictured waiting at the window was waiting for his grandchildren who were playing outside.”).

Results

**Base models.** As one of our original analysis techniques, we originally constructed a base model of the effects of interest, and then compared these base models to an additional model that also included math anxiety. We then used the anova function in R to compare the addition of math anxiety to the base model, allowing us to evaluate whether math anxiety accounted for a significant increase in the total amount of variance accounted for by each model. However, as we have chosen to focus on these models including math anxiety in the main manuscript, here we report the results for the base models and the model comparisons.

**Accuracy.** ***Base Model: Stimulus type x ER strategy.*** In the interest of focusing on the results that are most germane to our hypotheses, here we focused on the two categories of stimuli that were most relevant to math anxiety: analogy and mathematics. Discussion of results with all four categories of stimuli (math, analogy, negative, neutral) and can be found in the Supplementary Material. We first evaluated effects on accuracy using our base model: a LMM evaluating the effect of stimulus type (fixed effect, stimuli: analogy, math) and emotion regulation (fixed effect, emotion regulation: look, reappraise, suppress) on task accuracy was calculated across all participants (random effects accounting for individual differences across participants; REML criterion at convergence : -428). There was a main effect of stimulus type, *χ^2^*(1) = 4.13, *p* = .04, such that analogy trials (*M*_analogy_ = .722, *SD*_analogy_ = .11) had slightly lower accuracy than math trials (*M*_math_ = .746, *SD*_math_ = .13) across all participants. There was no main effect of emotion regulation strategy, *χ^2^*(2) = .17, *p* = .92, and no interaction, *χ^2^*(2) = 2.78, *p* = .25

***Model comparison.*** Finally, we compared the original Stimulus type x ER strategy LMM (base model) to the model that additionally added a measure of math anxiety as a fixed factor, to evaluate the whether the variance accounted for by the addition of math anxiety represents a measurable improvement in the model (using maximum likelihood estimation). When participants were grouped in the MA groups (MA groups, AIC: -463.76, BIC: -411.36) this represented an improvement compared to the base model (base model, AIC: -458.52, BIC: -428.57), *χ^2^*(6) = 17.24, *p* = .008. In short, the addition of math anxiety to the model accounting for stimulus type and ER strategy accounted for significantly more variance than the base model accounting for stimulus type and ER strategy alone; therefore, math anxiety significantly accounted for differences in task accuracy.

**Effects of Emotion Regulation on Electrodermal Activity.** In this analysis, we evaluated how EDA activity was related to math anxiety, stimulus type, and ER strategy. We evaluated increased sympathetic nervous system activity across stimulus categories and emotion regulation strategies. We calculated the integral (i.e., area under the curve) of the activity during the stimulus period, and z-scored the EDA values across all stimulus categories and emotion regulation strategies within each subject. Here we focused on EDA collected during the stimulus period (initial 5 seconds). As in the behavioral analyses, we analyzed the relationship between EDA as an outcome measure, and the variation associated with HMA vs. LMA individuals, stimulus type (math, analogy), and ER strategy (ES, CR, “Look”).

**Electrodermal Activity. *Base model: Stimulus type x ER strategy.*** We first examined EDA across all participants, using a LMM to evaluate a base model of the effects of stimulus type (fixed effect) and ER strategy (fixed effect) with each participant entered as a random effect, using EDA during the stimulus period as an outcome measure (REML criterion at convergence: 140.2). Across all participants, there was a trending main effect of stimulus type, *χ^2^*(1) = 3.25, *p* = .07, such that individuals have increased EDA during math trials (*M*_math_ = .05, *SD*_math_= .29), compared to analogy trials (*M*_analogy_ = -.005, *SD*_analogy_= .28). There was no main effect of ER strategy, *χ^2^*(2) = 1.07, *p* = .59, and no interaction between stimulus type and ER strategy, *χ^2^*(2) = 1.83, *p* = .40.

***EDA: Stimulus type x ER strategy x MA.*** We examined the effects on EDA of stimulus and ER strategy, and MA scores grouped on the basis of high and low MA controlling for trait anxiety (all fixed factors; random effects for each individual participant) using a LMM (REML criterion at convergence: 157.7). Importantly, and consistent with our hypotheses, we found a three-way interaction between MA group, stimulus type, and ER strategy, *χ^2^*(2) = 7.23, *p* = .03 (Supplementary Figure 2). This interaction is difficult to interpret, but we would like to draw attention to a couple of the specific effects that are of interest based on our hypotheses with regard to MA, emotion regulation strategy, and math. In order to evaluated some planned comparisons, we used post- hoc t-tests to make these comparisons. For HMA individuals, although we observed that the increased EDA during the control condition for math trials is reduced by CR and by ES, these comparisons between ER conditions are not statistically significantly different, all *p*’s > .60. EDA was not significantly elevated in the math control condition for HMA individuals compared to HMA individuals in the analogy condition, *t*(250) = -1.58, *p* = .12. Although we would expect that EDA is elevated in HMA individuals comparison to LMA individuals in the math control condition, this comparison was not statistically significant, *t*(300), = .96, *p* = .34. Although we observe a three-way interaction between MA, stimulus type, and ER strategy, it does not seem that these were driven by our specific comparisons of interest.

In the LMM exploring MA group, stimulus type, and emotion regulation, the main effect of stimulus type did not reach the threshold of significance (α = .05), *χ^2^*(1) = 3.71 , *p* = .054, and no main effect of ER strategy, *χ^2^*(2) = .92, *p* = .63, and no main effect of MA group, *χ^2^*(1) = .07, *p* = .78. There was no significant interaction in EDA between stimulus type and MA group, *χ^2^*(1) = .86, *p* = .35. There was no interaction between stimulus type and ER strategy, *χ^2^*(2) = 1.14, *p* = .56.

***Model comparison.*** In order to estimate the effect size of MA, we compared variance accounted for by MA groups to the base model of stimulus type x ER strategy for EDA as an outcome measure. When the model with MA groups (MA groups, AIC: 123.04, BIC: 175.44) is compared to the base model (base model, AIC: 120.87, BIC: 150.81), it does not account for a significant improvement in the amount of variance accounted for, *χ^2^*(6) = 9.83, *p* = .13.

***Base model: Stimulus type x ER strategy x EDA.*** In order to examine the association between arousal and behavioral performance, we first utilized a LMM with accuracy as the outcome measure. We examined the effect of arousal (EDA activity during the stimulus period), stimulus type, and ER strategy as fixed factors, and each individual accounted for by random effects (REML criterion at convergence: -397.8). Here we focused on accuracy as our measure of behavioral performance, because the results above were similar but less robust for reaction time. In this model, we found a significant effect of stimulus type, *χ^2^*(1) = 4.15, *p* = .04 (see previous accuracy results for explanation of these behavioral effects). There was no main effect of ER strategy, *χ^2^*(2) = .21, *p* = .90. There was no main effect of EDA, *χ^2^*(1) = .05, *p* = .83. There was no interaction between stimulus type and ER strategy on accuracy, *χ^2^*(2) = 2.32, *p* = .32. There was no interaction between stimulus type and EDA on accuracy, *χ^2^*(1) = .91, *p* = .34. There was no interaction between ER strategy and EDA on accuracy, *χ^2^*(2) = .41, *p* = .82. There was no three way interaction between stimulus type, ER strategy, and EDA on accuracy, *χ^2^*(2) = .12, *p* = .94.

***Model comparison.*** Comparing the models that include the additional factors of math anxiety to the base model (stimulus type, ER strategy, and EDA arousal), we found that the addition of math anxiety measures to these models accounts for significant additional variance. When HMA and LMA groups (controlling for trait anxiety; MA groups, AIC: -462.72, BIC: -365.40), *χ^2^*(12) = 38.53, *p* = .0001) are used in the model it accounts for more variance than the base model (base model, AIC: -448.19, BIC: -395.79). To conclude, the addition of math anxiety to these models accounting for the relationship between arousal and accuracy accounted for significantly more variance explained.

**Analyses including all 4 categories of stimuli.**

Here we discuss results utilizing all 4 categories of stimuli: math, analogy, negative pictures, and neutral pictures.

First we evaluated the fixed effects of stimulus type and emotion regulation on accuracy, with individual differences in intercept as a random effect. There was a main effect of stimulus type (fixed effect) on accuracy, *χ^2^*(3) = 15.49, *p* = .001, such that analogy trials (*M* = 72.2%, 95% CI: 69.8%-74.6%) were more difficult than math (*M* = 74.5%, *95% CI*: 72.1%-77.0%; *t*(561) = -1.95, *p* = .052), negative (*M* = 76.8%, *95% CI*: 74.4%-79.2%; *t*(561) = -3.80, *p* = .0002) and neutral (*M* = 75.6%, *95% CI*: 73.1%-77.9%; *t*(561) = -2.77, *p* = .006). There were no significant effects of emotion regulation technique, and no interaction between these fixed effects, *p* > .3. Across all participants, response accuracy in the negative condition was not influenced by emotion regulation.

We also evaluated how reactions to stimuli and emotion regulation instructions varied as a function of individual differences in anxiety. We hypothesized that math anxiety would interact with emotion regulation to influence the accuracy responses of HMA individuals to mathematics, compared to the other stimuli. In LMA individuals, we did not expect emotion regulation to influence responses in the math condition compared to the other conditions of stimuli. We used a linear mixed model to evaluate stimulus type, ER instructions and math anxiety group as mixed effects, with subject added as a random effect to account for individual differences. There was no main effect of ER, and no interactions with ER, all *p*s > .3. Again, we find a main effect of stimulus type on accuracy, *χ^2^*(3) = 15.13, *p* = .002, and an additional main effect of MARS group *χ^2^*(1) = 24.03, *p* < .001, such that the LMA group had higher accuracy overall (*M* = 78.6 %) than the HMA group (*M* = 70.6%). There was a trending interaction between stimulus type and math anxiety group, *χ^2^*(3) = 6.35, *p* = .095. When comparing these groups across stimuli, this interaction occurs with respect to math stimuli. For LMA individuals, accuracy in all conditions is elevated compared to the HMA group, but accuracy in the analogy condition is lower (*M*_LMA_ = 74.7%) than the accuracy observed in math (*M*_LMA_ = 79.9%), negative (*M*_LMA_ = 80.9%) and neutral (*M*_LMA_ = 78.8%). For HMA individuals, accuracy overall is lower, but a different pattern of results emerges: accuracy in the math condition (M_HMA_ = 68.7%) is lower that that in the analogy condition (M_HMA_ = 69.4%), whereas accuracy in both the negative (M_HMA_ = 72.4%) and neutral (M_HMA_ = 72.0%) is elevated in comparison, as in the LMA group. In other words, accuracy in the math condition was affected by level of math anxiety, and although math anxiety was associated with group differences in accuracy, the pattern of accuracy in the other categories of stimuli does not differ. All other interactions were not significant, all *p*’s > .3

*Reaction Time.* We also evaluated the effects of stimulus type and emotion regulation strategy on reaction time across all individuals. Again, because the salient nature of the negative stimuli, we expect that participants overall would show differential responses to the negative condition compared to the other stimuli. Similar to accuracy, we evaluated the fixed effects of stimulus type and emotion regulation on reaction time, with individual differences in intercept as a random effect. There was a main effect of stimulus type (fixed effect) on reaction time, such that analogy trials (*M* = 2350.69 ms, *95% CI*: 2254.95-2446.42 ms) had longer response times than math (*M* = 2057.26 ms, *95% CI*: 1961.52-2152.99 ms), negative (*M* = 2101.67 ms, *95% CI*: 2005.93-2197.40 ms) and neutral (*M* = 2073.71 ms; *95% CI*: 1977.98-2169.44 ms). There was a trending main effect of emotion regulation technique, *χ^2^*(2) = 5.85, *p* = .053. We observed longer response times in the reappraisal condition (*M* = 2183.22, *95% CI*: 2091.05-2275.39 ms) compared to the control condition (*M* = 2150.14, *95% CI*: 2057.97-2242.31 ms) and the suppression condition (*M* = 2104.13, *95% CI*: 2011.96-2196.30 ms). There was no interaction between stimulus type and emotion regulation technique, *p* > .3.

As an additional factor, we also evaluated how math anxiety group might influence interactions between stimulus type and emotion regulation strategy in reaction time. Again, there was a main effect of stimulus type, *χ^2^*(3) = 82.91, *p* < .0001, such that individuals responded more slowly to analogy trials. There was a trending effect of emotion regulation method, *χ^2^*(2) = 5.88, *p* = .053, again indicating that individuals responded more slowly to trials in the reappraisal condition overall. There was also a significant interaction between stimulus type and math anxiety group, *χ^2^*(3) = 23.52, *p* = .000032.

**Results for Math and Analogy Trials with Individual Differences in Math Anxiety**

**MA Group (groups controlling for trait anxiety, as in main manuscript).**

**Reaction Time.** ***Base model: Stimulus type x ER strategy.*** We first began by examining a base model that included the effects of stimulus type (fixed effect) and emotion regulation strategy (fixed effect) on reaction time across all participants (participants entered as a random effect) in a LMM (REML criterion at convergence: 4487). We find a main effect of stimulus type, *χ^2^*(1) = 75.14, *p* < .0001, such that analogy was associated with longer latencies (*M*_analogy_ = 2350.69 ms, *SD*_analogy_ = 438.59) than math (*M*_math_ = 2057.26 ms, *SD*_math_ = 402.34). There was also a main effect of emotion regulation strategy, *χ^2^*(2) = 6.41, *p* < .04, such that reappraise (*M*_reappraise_ = 2254.48, *SD*_reappraise_ = 456.45) had longer latencies than control (*M*_control_ = 2207.75, *SD*_control_ = 438.77), and especially longer than suppression (*M*_suppress_ = 2149.69, *SD*_suppress_ = 438.01). This extended processing time in the reappraisal condition was consistent with previous literature suggesting that reappraisal results in more elaborate and effortful processing of stimuli in concert with a reduction in negative affect (McRae et al., 2010; Spielberger, 2010). There was no significant interaction between stimulus type and emotion regulation strategy on reaction time, *χ^2^*(2) = .32, *p* = .85.

***MA model: Stimulus type x ER strategy x MA.*** When MA group was added as a fixed effect to our base model (see above for description; REML criterion at convergence: 4423.6), we again found a main effect of stimulus type, *χ^2^*(1) = 55.17, *p* < .0001(same as base model). There was not a significant main effect of ER strategy, *χ^2^*(2) = 4.11, *p* = .13. There was not a significant main effect of MA group, *χ^2^*(1) = .59, *p* = .44. There was not a significant interaction between stimulus type and ER strategy, *χ^2^*(2) = .90, *p* = .64. There was no significant interaction between MA group and ER strategy, *χ^2^*(2) = .16, *p* = .92. Similar to the results found for accuracy, we found a trending interaction between math anxiety group and stimulus type, *χ^2^*(1) = 2.93, *p* = .09, whereas the groups did not differ in the analogy condition, the HMA group has longer latencies than the LMA group in the math condition. There was no three way interaction between stimulus type, ER strategy, and MA group, *χ^2^*(2) = 1.53, *p* = .46. Across both accuracy and reaction time, we found a hypothesized deficit in mathematical performance associated with increased math anxiety, even when controlling for increased trait anxiety. This pattern of results indicated that math anxiety negatively influences both measures of performance across both tasks, and accuracy was most impacted for the math condition.

***Model comparison.*** When we compare the additional effects of math anxiety on reaction time to the base model (base model, AIC: 4552.9, BIC: 4582.8), adding MA group (MA group, AIC: 4559.5, BIC: 4611.9 ) does not account for significantly more variance than the base model, *χ^2^*(6) = 5.37, *p* = .50.

**Math Anxiety Groups (MARS Scores)**

*Accuracy*. Descriptive data for HMA and LMA groups are Supplementary Table 4. To examine the effects of math anxiety across stimulus types and emotion regulation conditions, groups based on the MARS (Suinn & Winston, 2003) were added to the base model as a fixed effect, so that the fixed effects were stimulus type, ER strategy, and MARS group, with random effects for participant (REML criterion at convergence: -406.5). Again, we find a marginally significant main effect of stimulus type, *χ^2^*(1) = 3.79, *p* = .051, such that analogy had lower accuracy than mathematics. We also find a main effect of MARS group, *χ^2^*(1) = 20.17, *p* < .0001, such that HMA individuals have substantially lower accuracy across tasks (*M*_HMA_ = .69, *SD*_HMA_ = .11) compared to LMA individuals (*M*_LMA_ = .77, *SD*_LMA_ = .12). Again there was no effect of ER strategy, *χ^2^*(2) = .15, *p* = .092. We also find a stimulus x math anxiety group interaction (Figure 2), *χ^2^*(1) = 6.35, *p* = .01, such that LMA individuals show increased accuracy in math compared to analogy, and HMA individuals do not have this advantage in the math condition. Instead, for HMA individuals, math performance is slightly lower than performance in analogy. This result replicates previous work illustrating that HMA individuals show a decrement in mathematics performance compared to LMA individuals. There was no interaction between stimulus type and ER strategy, *χ^2^*(2) = 2.76, *p* = .25, there was no interaction between ER strategy and MARS group, *χ^2^*(2) = 1.40, *p* = .50, and no three-way interaction between stimulus type, ER strategy, and MARS group, *χ^2^*(2) = .28, *p* = .86.

To estimate the effect size of MARS compared to the base model, we compared the original Stimulus type x ER strategy LMM (base model) to the models that additionally add measures of math anxiety as fixed factors, using the anova() function in R to evaluate the whether the variance accounted for by the addition of math anxiety represents a measurable improvement in the model (using maximum likelihood estimation). We find the addition of MARS groups (MARS group, AIC: -472.37, BIC: -419.96) improves the amount of variance accounted for compared to the base model (base model, AIC: -458.52, BIC: -428.57), *χ^2^*(6) = 25.85, *p* = .0002.

*Reaction Time.* When we examine these stimulus and ER effects with respect to math anxiety, we explored differences based on groups based on the MARS (fixed effect) in a LMM with reaction time as the outcome, adding this fixed effect to our base model (REML criterion at convergence: 4436.8). Similar to the results reported above, there was a main effect of stimulus type, *χ^2^*(1) = 73.60, *p* < .0001, and a main effect of emotion regulation strategy, *χ^2^*(2) = 6.38, *p* = .04, with effects mirroring those previously described. There was no significant effect of MARS group, *χ^2^*(1) = .21, *p* = .65. There was no interaction between stimulus type and ER strategy, *χ^2^*(2) = .33, *p* = .84. There was no interaction between MARS group and stimulus type, *χ^2^*(1) = .17, *p* = .68. There was no interaction between MARS group and ER strategy, *χ^2^*(2) = .24, *p* = .88. There was no three way interaction between MARS group, stimulus type, and ER strategy, *χ^2^*(2) = .64, *p* = .72.

When we compare the additional effects of math anxiety on reaction time to the base model (base model, AIC: 4552.9, BIC: 4582.8), we do not find that the addition of MARS group (MARS group, AIC: 4563.6, BIC: 4616.0) to this model accounts for significantly more variance than the base model, *χ^2^*(6) = 1.31, *p* = .97.

*Electrodermal Activity.* We examined EDA across stimulus type and ER strategy, adding MA groups (based on raw MARS scores) as a fixed factor to the base model using a LMM (REML criterion at convergence: 166.2). Again, we find a trending main effect of stimulus type, *χ^2^*(1) = 3.40, *p* = .06 (see previous). There was no main effect of ER strategy, *χ^2^*(2) = 1.06, *p* = .59. There was no main effect of MARS group, *χ^2^*(1) = .23, *p* = .63. There was no significant interaction between stimulus type and ER strategy for EDA, *χ^2^*(2) = 1.70, *p* = .43. There was no significant interaction between stimulus type and MARS group, *χ^2^*(1) = .64, *p* = .43. There was no significant interaction between ER strategy and MARS group, *χ^2^*(2) = 4.01, *p* = .13. We also find a trending interaction between MARS group, stimulus type, and ER strategy, *χ^2^*(2) = 4.60, *p* = .10. Whereas part of the interaction is driven by the differences in the suppression condition across math (*M_LMA_* = -.10, *SE_LMA_* = .05) and analogy (*M_LMA_* = .06, *SE_LMA_* = .05) for the LMA individuals, for HMA individuals, we observe increased arousal in the math condition in the control strategy (*M_HMA_* = .14, *SE_HMA_* = .06), and that this arousal is reduced in the math reappraisal strategy (*M_LMA_* = -.004, *SE_LMA_* = .06).

When we compare the addition of math anxiety as fixed factors to the base model of stimulus type x ER strategy for EDA as an outcome measure, we find that the addition of MARS groups (MARS groups, AIC: 123.16, BIC: 175.56) does not account for significantly more variance than the base model (base model, AIC: 120.87, BIC: 150.81), *χ^2^*(6) = 9.72, *p* = .14.

*Arousal and Behavior.* We examined these interactions in arousal and behavior with the additional effect of math anxiety. Looking at accuracy, we examined the effects of MARS groups in addition to our base model, stimulus type, ER strategy, and EDA, with random effects for each individual participant (REML criterion at convergence: -357.5). We find a weak main effect of stimulus type, *χ^2^*(1) = 2.78, *p* = .10. There was no main effect of ER strategy, *χ^2^*(2) = .10, *p* = .95. There was no main effect of EDA activity on accuracy, *χ^2^*(1) = .24, *p* = .62. There was a main effect of MARS group on accuracy, *χ^2^*(2) = 19.23, *p* < .0001 (LMA individuals have higher accuracy than HMA individuals across all tasks). We also find a two-way interaction between stimulus and MARS group on accuracy, *χ^2^*(1) = 6.59, *p* = .01. As before (see previous accuracy section), LMA individuals show increased accuracy in math compared to analogy, whereas HMA individuals do not show this advantage in math. There was no significant two-way interaction between stimulus type and ER strategy, *χ^2^*(2) = 2.88, *p* = .24. There was no interaction between ER strategy and EDA activity on accuracy, *χ^2^*(2) = 1.48, *p* = .48. There was no interaction between ER strategy and MARS group on accuracy, *χ^2^*(2) = 1.56, *p* = .46. We find a weak interaction between EDA and MARS group on accuracy, *χ^2^*(1) = 3.24, *p* = .07. There was no three way interaction between stimulus type, ER strategy and EDA on accuracy, *χ^2^*(2) = .15, *p* = .93, no three way interaction between stimulus type, ER method and MARS group, *χ^2^*(1) = 07, *p* = .95, no three way interaction between stimulus type, EDA, and MARS group, *χ^2^*(1) = 2.33, *p* = .13, and no three way interaction between ER strategy, EDA and MARS group on accuracy, *χ^2^*(2) = 2.69, *p* = .26. Finally, we find a trending 4-way interaction, between MARS group, EDA arousal during the stimulus period, stimulus type, and ER strategy, *χ^2^*(2) = 4.92, *p* = .09.

When MARS groups are added to the base model (base model, AIC: -448.19, BIC: -395.79), this model accounts for significantly more variance explained (w/MARS groups, AIC: -463.56, BIC: -366.24), *χ^2^*(12) = 39.36, *p* = .00009, than the base model.

**Results with Math Anxiety Controlling for Trait Anxiety (Continuous Measure)**

*Accuracy.* To further examine the effect of math anxiety on task accuracy, we also examined a measure of math anxiety while controlling for trait (general) anxiety, by using residuals from a regression with the STAI-trait subscale (Spielberger, 2010) predicting MARS scores (Suinn & Winston, 2003). In our sample, scores on the MARS are significantly correlated with trait anxiety (STAI), *r* = .55. In this way, we can examine the influence of specific math anxiety above and beyond the influence of broader patterns of anxiety. In a LMM with stimulus type (fixed effect), emotion regulation (fixed effect), and math anxiety controlling for trait (fixed effect) with random effects for each individual, we again find a main effect of stimulus type, *χ^2^*(1) = 4.06, *p* = .04, with a disadvantage for analogy compared to mathematics. There was also a main effect of math anxiety controlling for trait anxiety, such that increased math anxiety was associated with decreased performance across both tasks, *χ^2^*(1) = 21.81, *p* < .0001. However, we did not find any interactions between stimulus type and math anxiety when math anxiety was entered into the model as a continuous factor.

*Reaction Time.* Similarly, we examined the effect of math anxiety controlling for trait anxiety (MARS controlling for STAI-trait, continuous factor). Again, we find main effects of stimulus type *χ^2^*(1) = 74.46, *p* < .001, emotion regulation strategy, *χ^2^*(2) = 6.43, *p* = .04, and a main effect of math anxiety on reaction times across both categories *χ^2^*(1) = 4.10, *p* = .04, such that as math anxiety increases, RT increases (Supplementary Figure 1). Interestingly, this increasing effect of math anxiety on reaction time is perhaps counter to the speed-accuracy tradeoff, indicating increased reaction time and decreased accuracy on the basis of increased math anxiety. We believe that this effect indicates increased processing difficulty on the basis of increased math anxiety. Using MA controlling for trait anxiety scores as a fixed effect in the LMM, we do not find any significant interactions between any of the fixed factors, p > .3. Across both accuracy and response times, increased MA (controlling for trait anxiety) is associated with worse performance: slower response times and decreased accuracy. This pattern of results indicates that even controlling for trait anxiety, math anxiety negatively influences both measures of performance across both tasks, and accuracy is most impacted for the math condition.

*Electrodermal Activity (EDA).* We examined EDA across stimulus and ER strategy using math anxiety controlling for trait anxiety (MARS controlling for STAI-trait, continuous measure) in a LMM. Replicating the results from MARS scores, we find a trending main effect of stimuli *χ^2^*(1) = 3.37, *p* = .07. There was no main effect of ER strategy, *p* > .3. We find a significant three-way interaction between math anxiety (controlling for trait anxiety), stimulus type, and ER strategy, *χ^2^*(2) = 6.98, *p* = .03. Interestingly, when looking at MA along a continuous range, and controlling for trait anxiety, this interaction is largely driven by the analogy condition (Supplementary Figure 3). As math anxiety increases, reappraisal is associated with decreased skin conductance in the analogy condition, whereas the suppression condition results in a rebound of sympathetic activity (Gross, 1998; Gross & Levenson, 1993). In the math condition, we find that increased math anxiety is associated with increases in EDA across both control and reappraisal conditions, although the reappraisal condition seems to reduce this activity across the spectrum of math anxiety, suggesting subtle positive effects of the reappraisal strategy.

*Arousal and Behavior.* When we further examine these effects looking at math anxiety while controlling for trait anxiety (continuous scores), we find similar effects to those found with MARS groups. Again, we observe a main effect of stimulus type on accuracy, *χ^2^*(1) = 4.02, *p* = .05, a main effect of math anxiety (controlling for trait anxiety) on accuracy, *χ^2^*(2) = 21.09, *p* < .0001, and a trending interaction between EDA arousal and ER strategy on accuracy, *χ^2^*(2) = 4.84, *p* = .09. Finally, we find a similar 4-way interaction between math anxiety (MARS controlling for STAI), EDA arousal, stimulus type, and ER strategy, *χ^2^*(2) = 9.32, *p* = .001. All other interactions and main effects do not reach statistical significance, *p*’s > .14.

Supplementary Table 1. Simulation power analysis for accuracy predicted by MA groups, stimulus type, ER strategy.

| **Model:**  **Accuracy ~ Stimulus Type x ER strategy x MA group (random effects: participant)** | | | | | |
| --- | --- | --- | --- | --- | --- |
| **Fixed effects from model** | **Sample Size:**  **20** | **Sample Size:**  **30** | **Sample Size:**  **40** | **Sample Size:**  **50** | **Sample Size:**  **60** |
| Main Effect: Stimulus | 0.051 | 0.05 | 0.052 | 0.057 | 0.044 |
| Main Effect: ER strategy (Control vs. Reappraise) | 0.053 | 0.045 | 0.046 | 0.043 | 0.034 |
| Main Effect: ER strategy (Control vs. Suppress) | 0.045 | 0.042 | 0.051 | 0.045 | 0.048 |
| Main effect: MA groups* | 0.629 | 0.791 | 0.898 | 0.953 | 0.979 |
| Interaction: Stimulus x ER Strategy (Control vs. Reappraise) | 0.08 | 0.085 | 0.089 | 0.104 | 0.123 |
| Interaction: Stimulus x ER Strategy (Control vs. Suppress) | 0.057 | 0.05 | 0.049 | 0.04 | 0.056 |
| Interaction: Stimulus x MA groups* | 0.254 | 0.291 | 0.4 | 0.48 | 0.558 |
| Interaction: ER Strategy (Control vs Reappraise) x MA groups | 0.057 | 0.054 | 0.04 | 0.041 | 0.047 |
| Interaction: ER Strategy (Control vs Suppress) x MA groups | 0.045 | 0.046 | 0.05 | 0.053 | 0.061 |
| Interaction: Stimulus x ER Strategy (Control vs. Reappraise) x MA groups | 0.055 | 0.058 | 0.052 | 0.063 | 0.081 |
| Interaction: Stimulus x ER Strategy (Control vs. Suppress) x MA groups | 0.05 | 0.062 | 0.049 | 0.046 | 0.053 |

Note: Simulations (1000 iterations) used to estimate statistical power based on the present dataset. Simulations used estimated sample size to calculate the proportion of estimated statistically significant simulations compared to the total number of simulations to calculate estimated statistical power. Effects that are marked with * were effects that were considered statistically significant at the *p* < .05 level in our dataset, effects marked with ^ are trending, .05 < *p* < .10 (N = 52).

Supplementary Table 2. Simulation power analysis for EDA predicted by MA groups, stimulus type, ER strategy.

| **Model:**  **EDA ~ Stimulus Type x ER strategy x MA group (random effects: participant)** | | | | | |
| --- | --- | --- | --- | --- | --- |
| **Fixed effects from model** | **Sample Size:**  **20** | **Sample Size:**  **30** | **Sample Size:**  **40** | **Sample Size:**  **50** | **Sample Size:**  **60** |
| Main Effect: Stimulus* | 0.235 | 0.306 | 0.383 | 0.469 | 0.519 |
| Main Effect: ER strategy (Control vs. Reappraise) | 0.078 | 0.108 | 0.122 | 0.144 | 0.17 |
| Main Effect: ER strategy (Control vs. Suppress) | 0.046 | 0.055 | 0.055 | 0.054 | 0.06 |
| Main effect: MA groups | 0.031 | 0.04 | 0.045 | 0.053 | 0.043 |
| Interaction: Stimulus x ER Strategy (Control vs. Reappraise) | 0.042 | 0.053 | 0.054 | 0.04 | 0.065 |
| Interaction: Stimulus x ER Strategy (Control vs. Suppress) | 0.077 | 0.126 | 0.144 | 0.192 | 0.216 |
| Interaction: Stimulus x MA groups | 0.091 | 0.103 | 0.142 | 0.162 | 0.154 |
| Interaction: ER Strategy (Control vs Reappraise) x MA groups | 0.093 | 0.104 | 0.111 | 0.124 | 0.159 |
| Interaction: ER Strategy (Control vs Suppress) x MA groups | 0.097 | 0.09 | 0.092 | 0.098 | 0.136 |
| Interaction: Stimulus x ER Strategy (Control vs. Reappraise) x MA groups* | 0.052 | 0.082 | 0.083 | 0.079 | 0.086 |
| Interaction: Stimulus x ER Strategy (Control vs. Suppress) x MA groups* | 0.374 | 0.512 | 0.629 | 0.715 | 0.808 |

Note: Simulations (1000 iterations) used to estimate statistical power based on the present dataset. Simulations used estimated sample size to calculate the proportion of estimated statistically significant simulations compared to the total number of simulations to calculate estimated statistical power. Effects that are marked with * were effects that were considered statistically significant at the *p* < .05 level in our dataset, effects marked with ^ are trending, .05 < *p* < .10 (N = 52).

Supplementary Table 3. Simulation power analysis for EDA predicted by MA groups, stimulus type, ER strategy.

| **Model:**  **Accuracy ~ Stimulus Type x ER strategy x MA group x EDA (random effects: participant)** | | | | | |
| --- | --- | --- | --- | --- | --- |
| **Fixed effects from model** | **Sample Size:**  **20** | **Sample Size:**  **30** | **Sample Size:**  **40** | **Sample Size:**  **50** | **Sample Size:**  **60** |
| Main Effect: Stimulus | 0.06 | 0.047 | 0.051 | 0.043 | 0.047 |
| Main Effect: ER strategy (Control vs. Reappraise) | 0.058 | 0.034 | 0.052 | 0.064 | 0.062 |
| Main Effect: ER strategy (Control vs. Suppress) | 0.048 | 0.041 | 0.043 | 0.04 | 0.044 |
| Main effect: MA groups* | 0.578 | 0.742 | 0.839 | 0.918 | 0.965 |
| Main effect: EDA | 0.064 | 0.052 | 0.063 | 0.06 | 0.078 |
| Interaction: Stimulus x ER Strategy (Control vs. Reappraise) | 0.064 | 0.072 | 0.095 | 0.107 | 0.121 |
| Interaction: Stimulus x ER Strategy (Control vs. Suppress) | 0.061 | 0.07 | 0.066 | 0.076 | 0.084 |
| Interaction: Stimulus x MA groups^ | 0.172 | 0.252 | 0.312 | 0.403 | 0.416 |
| Interaction: ER Strategy (Control vs Reappraise) x MA groups | 0.06 | 0.062 | 0.049 | 0.061 | 0.063 |
| Interaction: ER Strategy (Control vs Suppress) x MA groups | 0.059 | 0.054 | 0.05 | 0.044 | 0.071 |
| Interaction: Stimulus x EDA | 0.125 | 0.158 | 0.2 | 0.279 | 0.324 |
| Interaction: EDA x ER Strategy (Control vs. Reappraise) | 0.077 | 0.085 | 0.086 | 0.127 | 0.136 |
| Interaction: EDA x ER Strategy (Control vs. Suppress) | 0.048 | 0.052 | 0.049 | 0.044 | 0.035 |
| Interaction: MA x EDA | 0.05 | 0.074 | 0.057 | 0.064 | 0.09 |
| Interaction: Stimulus x ER Strategy (Control vs. Reappraise) x MA groups | 0.046 | 0.054 | 0.073 | 0.065 | 0.078 |
| Interaction: Stimulus x ER Strategy (Control vs. Suppress) x MA groups | 0.072 | 0.091 | 0.118 | 0.135 | 0.151 |
| Interaction: Stimulus x ER Strategy (Control vs. Reappraise) x EDA^ | 0.131 | 0.194 | 0.224 | 0.312 | 0.34 |
| Interaction: Stimulus x ER Strategy (Control vs. Suppress) x EDA^ | 0.177 | 0.266 | 0.339 | 0.437 | 0.449 |
| Interaction: Stimulus x MA groups x EDA | 0.132 | 0.197 | 0.268 | 0.33 | 0.386 |
| Interaction: ER Strategy (Control vs. Reappraise) x MA groups x EDA | 0.039 | 0.049 | 0.045 | 0.06 | 0.044 |
| Interaction: ER Strategy (Control vs. Suppress) x MA groups x EDA | 0.152 | 0.208 | 0.272 | 0.352 | 0.391 |
| Interaction: Stimulus x ER Strategy (Control vs. Reappraise) x MA groups x EDA* | 0.263 | 0.429 | 0.551 | 0.677 | 0.722 |
| Interaction: Stimulus x ER Strategy (Control vs. Suppress) x MA groups x EDA* | 0.405 | 0.628 | 0.768 | 0.868 | 0.899 |

Note: Simulations (1000 iterations) used to estimate statistical power based on the present dataset. Simulations used estimated sample size to calculate the proportion of estimated statistically significant simulations compared to the total number of simulations to calculate estimated statistical power. Effects that are marked with * were effects that were considered statistically significant at the *p* < .05 level in our dataset, effects marked with ^ are trending, .05 < *p* < .10 (N = 52).

Supplementary Table 4. Descriptive statistics for HMA and LMA Groups.

|  | **ALL** | **MARS GROUPS** | |
| --- | --- | --- | --- |
| **Measure (Mean (SD))** | **All (N = 52)** | **LMA**  **(*n* = 27)** | **HMA**  **(*n* = 25)** |
| *Age* | 19.56 (1.15) | 19.70 (1.27) | 19.4 (1.0) |
| *Gender --% Female* | 63.6 % | 44.4 % | 84 % |
| *Math Anxiety Rating Scale*  *(MARS)* | 2.26 (.81) | 1.53 (.22) | 3.06 (.30) |
| *Math Anxiety Controlling for Test Anxiety (MA-STAI)* | -.003 (.65) | -.50 (.39) | .53 (.48) |
| *Spielberger Test Anxiety Inventory (TAI)* | 1.91 (.65) | 1.54 (.44) | 2.30 (.62) |
| *Spielberger State-Trait Anxiety Inventory—Trait subscale (STAI)* | 2.06 (.50) | 1.78 (.40) | 2.36 (.41) |
| *Emotion Regulation Questionnaire- Cognitive Reappraisal subscale*  *(ERQ—CR)* | 5.12 (.90) | 5.32 (.72) | 4.19 (1.03) |
| *Emotion Regulation Questionnaire- Expressive Suppression subscale*  *(ERQ—ES)* | 2.87 (1.02) | 3.13 (1.03) | 2.60 (.95) |
| *Positive and Negative Affect Schedule—Negative Affect*  *(PANAS—NA)* | 1.94 (.70) | 1.61 (.46) | 2.3 (.74) |
| *Positive and Negative Affect Schedule—Positive Affect*  *(PANAS—PA)* | 3.45 (.60) | 3.56 (.54) | 3.33 (.66) |
| *Writing Anxiety (WA)* | 2.81 (.67) | 2.83 (.70) | 2.8 (.66) |

Note. Summary statistics for low and high MA groups from MARS Scores.

Supplementary Figure 1. The influence of math anxiety (controlling for trait anxiety) on response times across conditions.

Note. As math anxiety controlling for trait anxiety (MARS controlling for STAI-trait) increases, response times increase across both stimulus conditions, *χ^2^*(1) = 4.10, *p* = .04.

*Supplmentary Figure 2.* Interaction between math anxiety, stimulus type and emotion regulation strategy in electrodermal activity.

Note. Electrodermal activity during the stimulus period shows an interaction between math anxiety, stimulus type and emotion regulation strategy, *χ^2^*(2) = 7.23, *p* = .03. Here the interaction is depicted on the basis of math anxiety groups controlling for trait anxiety. For HMA individuals we observe that the increased EDA during the control condition for math trials is reduced by CR and by ES. In contrast, the HMA group shows no increased arousal levels during the analogy trials. Overall, the LMA group shows different patterns of EDA, especially during the ES conditions and the control condition for math trials.

*Supplementary Figure 3*. Interactions between math anxiety, stimulus type and emotion regulation strategy in electrodermal activity.

Note. Electrodermal activity during the stimulus period shows an interaction between math anxiety, stimulus type and emotion regulation strategy and MA scores controlling for trait anxiety on a continuous scale.
